# Critical and differential roles of eIF4A1 and eIF4A2 in B-cell development and function

**DOI:** 10.1038/s41423-024-01234-x

**Published:** 2024-11-08

**Authors:** Ying Du, Jun Xie, Dewang Liu, Jiayi Zhao, Pengda Chen, Xiaoyu He, Peicheng Hong, Yubing Fu, Yazhen Hong, Wen-Hsien Liu, Changchun Xiao

**Affiliations:** 1 State Key Laboratory of Cellular Stress Biology, School of Life Sciences, Faculty of Medicine and Life Sciences, Xiamen University, Xiamen, Fujian 361102 China; 2 Department of Immunology and Microbiology, The Scripps Research Institute, La Jolla, CA 92037 USA; 3 Present Address: Sanofi Institute for Biomedical Research, Suzhou, Jiangsu 215123 China

**Keywords:** RNA helicase, eIF4A, Ribosome biogenesis, Translation, B cell, Humoral immunity, Epigenetics in immune cells

## Abstract

Eukaryotic initiation factor 4 A (eIF4A) plays critical roles during translation initiation of cellular mRNAs by forming the cap-binding eIF4F complex, recruiting the 40S small ribosome subunit, and scanning the 5’ untranslated region (5’ UTR) for the start codon. eIF4A1 and eIF4A2, two isoforms of eIF4A, are highly conserved and exchange freely within eIF4F complexes. The understanding of their biological and molecular functions remains incomplete if not fragmentary. In this study, we showed that eIF4A1 and eIF4A2 exhibit different expression patterns during B-cell development and activation. Mouse genetic analyses showed that they play critical but differential roles during B-cell development and humoral immune responses. While eIF4A1 controls global protein synthesis, eIF4A2 regulates the biogenesis of 18S ribosomal RNA and the 40S ribosome subunit. This study demonstrates the distinct cellular and molecular functions of eIF4A1 and eIF4A2 and reveals a new role of eIF4A2 in controlling 40S ribosome biogenesis.

## Introduction

In mammalian cells, translation initiation starts with the assembly of the eukaryotic initiation factor 4 F (eIF4F) complex, which consists of the cap-binding protein eIF4E, the scaffold protein eIF4G, and the RNA helicase eIF4A, on the 5’ cap of messenger RNAs (mRNAs). A ternary complex including eIF2F, GTP, and methionyl-initiator transfer RNA (Met-tRNAi), together with several initiation factors (eIF1, eIF1A, eIF3, and eIF5), joins the 40S ribosome subunit to form a 43S preinitiation complex (PIC). The 43S PIC is then recruited to the 5’ cap structure by the eIF4F complex. This mRNA-bound PIC scans the 5’ untranslated region (5’UTR) until the start codon (usually AUG) is encountered, whereupon the 60S ribosome subunit is recruited to form the 80S ribosome. Next, GTP is hydrolyzed, eIF5B and eIF1A are discharged, and the newly assembled 80S ribosome is now poised for translation elongation [1].

eIF4A is an ATP-dependent RNA helicase of the DEAD-box family that unwinds secondary structures in the 5’ UTR of mRNAs. This unwinding is essential for the PIC to scan the 5’UTR to find the start codon. Mammals possess two highly related eIF4A paralogs, eIF4A1 and eIF4A2, which in humans are 90% identical at the amino acid level. Evolutionarily, eIF4A1 and eIF4A2 start to diverge beyond *C. elegans*, and high conservation of eIF4A2 is observed only in vertebrates. eIF4A1 and eIF4A2 have been shown to exchange freely within eIF4F complexes in vitro, and owing to their similarity, it has long been thought that they are functionally interchangeable [2]. However, new evidence has emerged suggesting that the expression of eIF4A1 and eIF4A2 is differentially regulated in a tissue-specific fashion and under various cell growth conditions. Moreover, recent evidence suggests that eIF4A1 and eIF4A2 may be functionally distinct in vivo [3–5].

Mouse genetic studies focusing on eIF4A1 and eIF4A2 have provided valuable insights into the biological functions of these two eukaryotic initiation factors. Germline knockout of eIF4A1 caused embryonic lethality, indicating that eIF4A1 performs critical functions necessary for early development. In vitro studies with cells derived from these mutant mice confirmed the crucial role of eIF4A1 in supporting the basic translational machinery needed for cellular growth and proliferation. In contrast, mutant mice with germline deletion of eIF4A2 are viable, indicating that it is not essential for embryonic development or immediate postnatal survival. This finding suggests that the functions of eIF4A2 might be more specialized or that there is functional redundancy with other proteins that can compensate for its absence [3]. Further investigations, especially conditional knockout studies targeting specific cell types, tissues, or developmental stages, will be crucial for unraveling the specific functions and molecular mechanisms of action of eIF4A1 and eIF4A2 in animals.

We study the functions and mechanisms of action of RNA helicases in the mammalian immune system, with a focus on B cells [6]. B cells are a vital component of the mammalian immune system and play critical roles in our body’s defense against infections and diseases. Upon exposure to appropriate stimuli, B cells increase in size and start to proliferate. Activated B cells either directly differentiate into plasma cells, which produce antibodies of relatively low affinity, or re-enter the B-cell follicle and, with the help of a distinct CD4^+^ effector T-cell subset named T follicular helper (T_FH_) cells, form germinal centers (GCs). B cells in the GC (GCB) undergo extensive proliferation, class-switch recombination, and somatic hypermutation. The GC response ultimately produces high-affinity, long-lived plasma cells capable of sustaining a high level of antibody secretion and memory B cells that maintain the B-cell phenotype but are programmed to rapidly differentiate into plasma cells upon encountering the same antigen [7–9].

B cells exhibit remarkable complexity in their regulation and function. Posttranscriptional regulation in B cells involves a broad range of players and molecular processes, including RNA-binding proteins (RBPs) [10–12], microRNAs (miRNAs) [13–15], alternative splicing [16], RNA editing [17], and posttranslational modifications [18]. These mechanisms collectively govern gene expression, mRNA stability, and protein synthesis, allowing B cells to fine-tune their responses to environmental cues. Dysregulation of B cells causes autoimmune diseases and lymphoma. Expression of eIF4A1 is significantly upregulated in diffuse large B-cell lymphoma (DLBCL), which is predictive of poor patient prognosis [19]. eIF4A inhibition leads to positive outcomes in B-cell lymphoma [19] and leukemia [20]. While the roles of eIF4A in general protein synthesis and cancer biology are well established, its specific functions in B cells are not thoroughly understood. The precise regulation of protein synthesis is vital for B-cell development and antibody production, leaving a knowledge gap in understanding how eIF4A affects B-cell biology. These findings could provide insights into translational control during immune responses and identify new therapeutic targets for immune-related disorders.

In this study, we generated conditional knockout alleles of eIF4A1 and eIF4A2, deleted them at different stages of B-cell development, and investigated their functions and molecular mechanisms of actions. Our findings show that they exhibit critical but distinct cellular and molecular functions and discover a new role of eIF4A2 in controlling the biogenesis of 18S ribosomal RNA and the 40S ribosome subunit.

## Results

### eIF4A1 and eIF4A2 play critical roles during early B-cell development

**Fig. 1:**
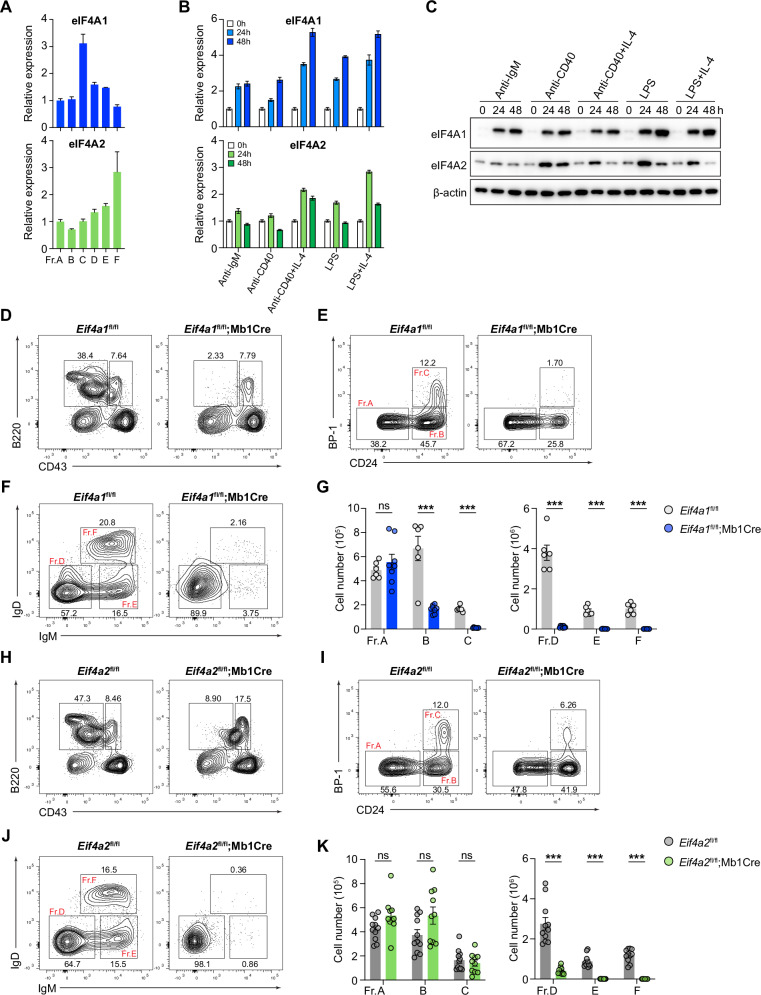
**eIF4A1 and eIF4A2 are required for early B cell development**. **A** RT‒qPCR analysis of eIF4A1 and eIF4A2 mRNA expression in bone marrow B lineage cells. **B, C** RT‒qPCR **B** and immunoblot **C** analysis of eIF4A1 and eIF4A2 mRNA and protein expression in naïve B cells activated with various stimuli for the indicated amounts of time. **D**–**K** Flow cytometry analysis of B lineage cells in the bone marrow of *Eif4a1*^fl/fl^, *Eif4a1*^fl/fl^;*Mb1Cre* **D**–**G** and *Eif4a2*^fl/fl^, *Eif4a2*^fl/fl^;*Mb1Cre* **H**–**K** mice. **D, E, F, H, I, J** Representative FACS plots. **G, K** Bar graphs summarizing cell numbers. Each symbol represents an individual mouse. The error bars represent the standard errors of the means (SEMs). ns, not significant; *p < 0.05, **p < 0.01 and ***p < 0.001. The data shown are representative of three independent experiments. In **G**, **K**, the data represent the combination of two independent experiments

We first examined the expression of eIF4A1 and eIF4A2 during B-cell development in the bone marrow and in mature B cells activated by various stimuli mimicking B-cell receptor stimulation (anti-IgM), T-cell help (anti-CD40 and anti-CD40 + IL-4), and T-cell-independent signaling (LPS and LPS + IL-4). As shown in Fig. 1A–C, eIF4A1 mRNA expression was high in Fraction C (Fr. C), diminished afterwards, and strongly induced upon B cell activation. In contrast, eIF4A2 mRNA expression gradually increased during B cell development and reached the highest level in mature B cells recirculated back to the bone marrow (Fr.F). Upon B cell activation, eIF4A2 mRNA and protein expression was upregulated to various degrees by 24 h but decreased by 48 h (Fig. 1B, C). The dynamic changes in the expression of eIF4A1 and eIF4A2 during B-cell development and activation suggest important roles of this family in B cells. To investigate the biological function of eIF4A1 and eIF4A2, we generated loxP-site flanked alleles of these two genes (Supplementary Fig. 1A–D) and crossed the mutant mice with *Mb1*Cre mice in which the *CD79a* gene was replaced with a codon-optimized Cre recombinase gene [21]. The *CD79a* locus turns on Cre expression and drives efficient deletion of loxP site-flanking alleles at an early developmental stage of the B-cell lineage. Both *Eif4a1*^*fl/fl*^*;Mb1Cre* and *Eif4a2*^*fl/fl*^*;Mb1Cre* mice appeared healthy but presented few B cells in the spleen and peripheral lymph nodes (Supplementary Fig. 2). Loss of peripheral B cells may be caused by defective B-cell development. Thus, we examined B-cell development in the bone marrow. Consistent with high eIF4A1 expression in Fr.C. (Fig. 1A), *Eif4a1*^*fl/fl*^*;Mb1Cre* mice presented a progressive and drastic decrease in the number of Fr.B. and Fr.C. cells and a complete lack of B lineage cells after Fr.C. (Fig. 1D–G). *Eif4a2*^*fl/fl*^*;Mb1Cre* mice exhibited similar impairments in B cell development, but the decrease did not manifest until the Fr.D, accompanied by a slight reduction in the percentage of Fr.C cells (Fig. 1H–K). Therefore, both eIF4A1 and eIF4A2 play critical roles during early B cell development, but at different developmental stages.

### eIF4A1 and eIF4A2 are indispensable for the T-cell-dependent antibody response

The lack of peripheral B cells in *Eif4a1*^*fl/fl*^*;Mb1Cre* and *Eif4a2*^*fl/fl*^*;Mb1Cre* mice prevented the study of the immune functions of eIF4A1 and eIF4A2. Therefore, we crossed *Eif4a1*^*fl/fl*^ and *Eif4a2*^*fl/fl*^ mice with *CD19Cre* mice, in which expression of the Cre recombinase is under the control of the *CD19* locus [22, 23]. Cre expression is turned on at Frs. B and C in *CD19Cre* mice, with a deletion efficiency of about 33% in bone marrow B cells and 90–97% in mature B cells of the periphery [22, 23]. This allows bypassing of early B-cell developmental defects in *Eif4a1*^*fl/fl*^*;Mb1Cre* and *Eif4a2*^*fl/fl*^*;Mb1Cre* mice. B-cell development was normal in *Eif4a1*^*fl/fl*^*;CD19Cre* and *Eif4a2*^*fl/fl*^*;CD19Cre* mice. The proportions and numbers of various B-cell subsets in the spleen and peripheral lymph nodes of *Eif4a1*^*fl/fl*^*;CD19Cre* and *Eif4a2*^*fl/fl*^*;CD19Cre* mice were also similar to that of wild type mice (Supplementary Fig. 3).

**Fig. 2:**
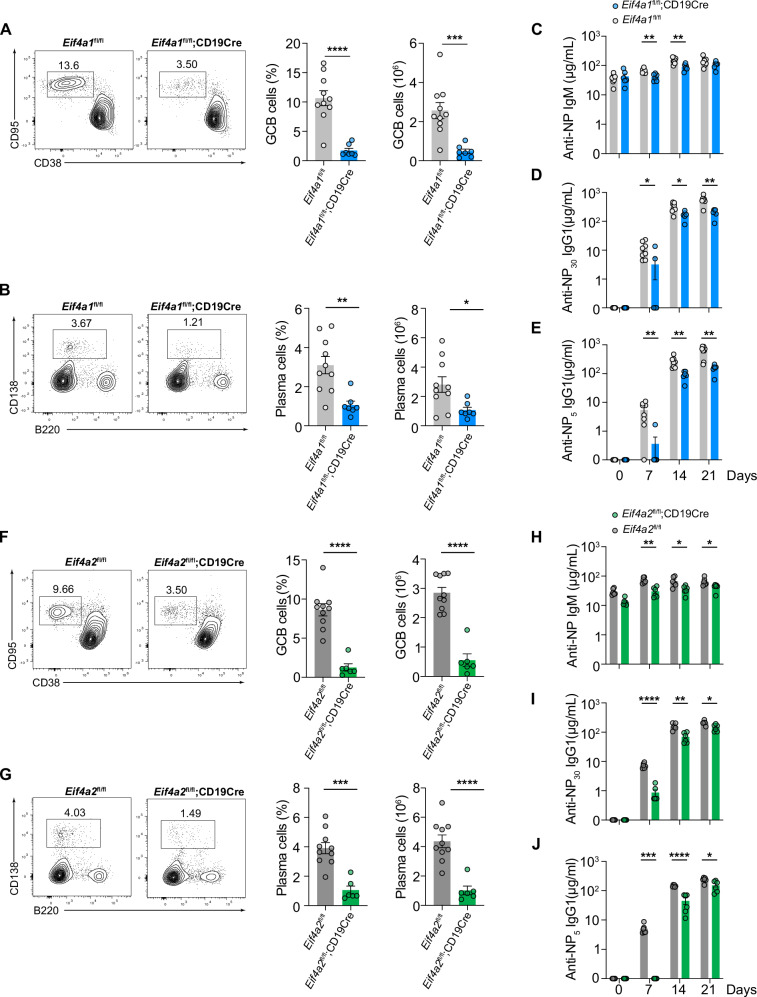
**eIF4A1 and eIF4A2 are indispensable for the T-cell-dependent B cell response**. **A,** **B, F, G** Flow cytometry analysis of GCB (CD38^-^CD95^+^) **A, F** and plasma cells (B220^low/-^CD138^+^) **B, G** in the spleens of *Eif4a1*^fl/fl^ (*n* = 10), *Eif4a1*^fl/fl^;CD19Cre (*n* = 7) **A,** **B** and *Eif4a2*^fl/fl^ (*n* = 10), and *Eif4a2*^fl/fl^;CD19Cre (*n* = 6) **F,** **G** mice on day 7.5 after immunization with OVA/alum/LPS. Left, representative FACS plots; right, summary of the percentage and number of GCB and plasma cells. **C, D, E, H, I, J** *Eif4a1*^fl/fl^ (*n* = 8), *Eif4a1*^fl/fl^;CD19Cre (*n* = 6), *Eif4a2*^fl/fl^ (*n* = 7), and *Eif4a2*^fl/fl^;CD19Cre (*n* = 6) mice were immunized with NP-OVA/Alum. Serum concentrations of NP-specific IgM **C, H** and IgG1 **D,** **E, I, J** antibodies at the indicated time points were determined by ELISA. Each symbol represents an individual mouse. The error bars represent the standard errors of the means (SEMs). ns, not significant; *p < 0.05, **p < 0.01 and ***p < 0.001. The data shown are representative of three independent experiments

To investigate the roles of eIF4A1 and eIF4A2 in the humoral immune response, *Eif4a*1^fl/fl^;*CD19Cre* and *Eif4a2*^*fl/fl*^*;CD19Cre* mice were immunized with ovalbumin (OVA) precipitated in alum accompanied with lipopolysaccharide (LPS) (termed OVA/alum/LPS). Flow cytometry analysis of splenocytes from immunized mice showed that the percentage and number of GCB and plasma cells were significantly reduced in *Eif4a*1^fl/fl^;*CD19Cre* and *Eif4a*2^fl/fl^;*CD19Cre* mice (Fig. 2A, B, F, G). Furthermore, we examined the antigen-specific antibody response by immunizing *Eif4a*1^fl/fl^;*CD19Cre* and *Eif4a*2^fl/fl^;*CD19Cre* mice with 4-hydroxy-3-nitrophenyl hapten conjugated to ovalbumin (NP-OVA) precipitated in alum (termed NP-OVA/alum). As shown in Fig. 2C–E, H–J, NP-specific total IgG1 (anti-NP_30_ IgG1) and high-affinity IgG1 (anti-NP_5_ IgG1) were drastically decreased in both *Eif4a*1^fl/fl^;*CD19Cre* and *Eif4a*2^fl/fl^;*CD19Cre* mice, accompanied by a slight reduction in NP-specific IgM antibodies. Taken together, our results demonstrate that both eIF4A1 and eIF4A2 are indispensable for the T-cell-dependent B-cell response, including GCB formation, plasma cell differentiation, and antibody production.

### Differential roles of eIF4A1 and eIF4A2 in T-cell-independent antibody responses

**Fig. 3:**
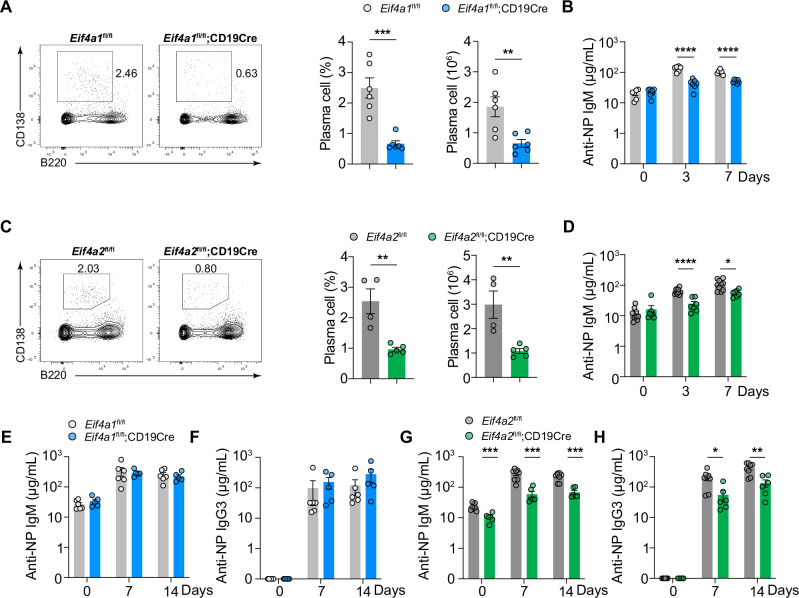
**eIF4A2 controls both the TI-1 and TI-2 B-cell responses, whereas eIF4A1 is required for the TI-1 response**. **A, C** Flow cytometry analysis of plasma cells in the spleens of *Eif4a1*^fl/fl^ (*n* = 6), *Eif4a1*^fl/fl^;CD19Cre (*n* = 6) **A** and *Eif4a2*^fl/fl^ (*n* = 4), and *Eif4a2*^fl/fl^;CD19Cre (*n* = 5) **C** mice on day 3 after NP‒LPS immunization. Left, representative FACS plots; right, summary of the percentage and number of plasma cells. **B, D** Serum concentrations of the NP-specific IgM antibody were determined via ELISA at the indicated time points after NP‒LPS immunization in *Eif4a1*^fl/fl^ (*n* = 6), *Eif4a1*^fl/fl^;CD19Cre (*n* = 7) **B**, *Eif4a2*^fl/fl^ (*n* = 10), and *Eif4a2*^fl/fl^;CD19Cre (*n* = 7) **D** mice. **E–H** ELISA analysis of NP-specific IgM **E, G** and IgG3 **F, H** in *Eif4a1*^fl/fl^ (*n* = 6), *Eif4a1*^fl/fl^;CD19Cre (*n* = 5) **E, F** and *Eif4a2*^fl/fl^ (*n* = 8), and *Eif4a2*^fl/fl^;CD19Cre (*n* = 6) **G, H** mice after NP-Ficoll immunization. Each symbol represents an individual mouse. The error bars represent the standard errors of the means (SEMs). ns not significant; *p < 0.05, **p < 0.01 and ***p < 0.001. The data shown are representative of three independent experiments

We further investigated the roles of eIF4A1 and eIF4A2 in T-cell-independent (TI) antibody responses. *Eif4a1*^*fl/fl*^*;CD19Cre* and *Eif4a2*^*fl/fl*^*;CD19Cre* mice were immunized with 4-hydroxy-3-nitrophenyl hapten-conjugated LPS (NP-LPS), a type 1 TI (TI-1) antigen that activates B cells through both the NP-specific B-cell receptor (BCR) and Toll-like receptor 4 (TLR4). As shown in Fig. 3A–D, B-cell-specific deletion of either eIF4A1 or eIF4A2 resulted in drastic reductions in the percentage and number of plasma cells, as well as the titers of NP-specific IgM antibodies. We then immunized these mice with NP-Ficoll, a type 2 TI (TI-2) antigen that consists of a highly repetitive surface structure (NP) conjugated to a polysaccharide (Ficoll). NP-Ficoll activates NP-specific B cells by crosslinking many BCRs, resulting in proliferation, plasma cell differentiation, and antibody production. While B-cell-specific deletion of eIF4A1 had no obvious effect on the production of NP-specific IgM and IgG3 antibodies (Fig. 3E, F), deletion of eIF4A2 caused significant reductions in both NP-specific IgM and IgG3 antibodies (Fig. 3G, H). Therefore, eIF4A1 is required only for the TI-1 antibody response, while eIF4A2 is required for both the TI-1 and TI-2 antibody responses.

### eIF4A1 and eIF4A2 control B cell proliferation

**Fig. 4:**
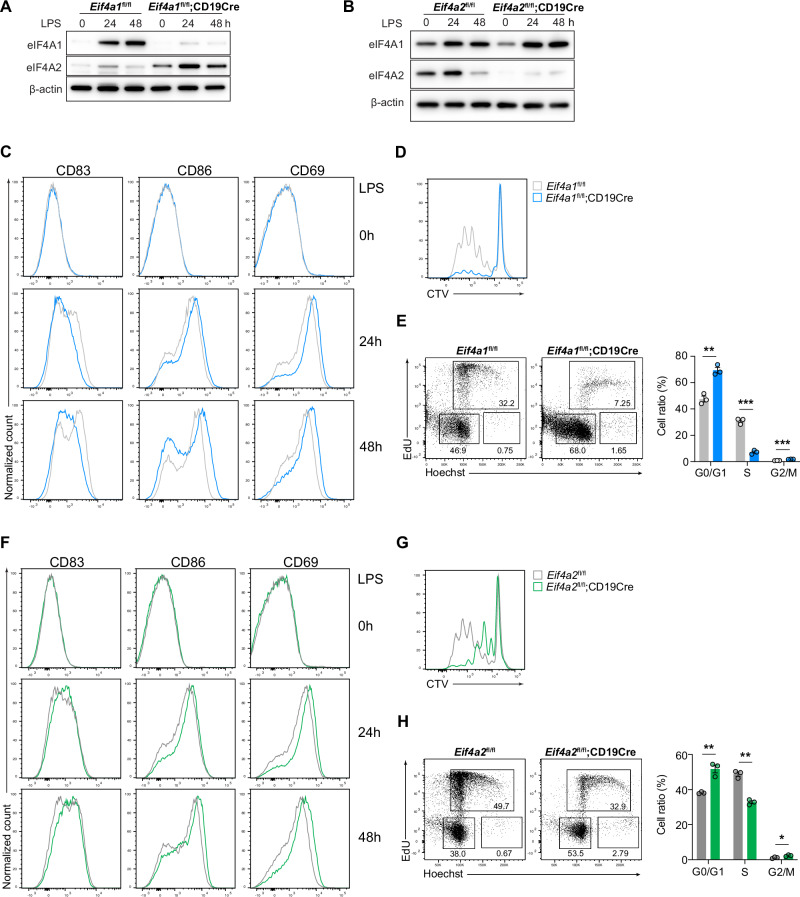
**eIF4A1 and eIF4A2 control B cell proliferation**. **A, B** Immunoblot analysis of eIF4A1 and eIF4A2 protein expression in B cells purified from *Eif4a1*^fl/fl^, *Eif4a1*^fl/fl^;CD19Cre **A**, *Eif4a2*^fl/fl^*, and Eif4a2*^fl/fl^;CD19Cre **B** mice and stimulated with LPS for the indicated amounts of time. **C, F** Flow cytometry analysis of activation marker expression on B cells stimulated with LPS for the indicated amounts of time. **D, G** B cells were labeled with CellTrace Violet (CTV), stimulated with LPS for 3 days, and analyzed by flow cytometry for cell division. **E, H** B cells were stimulated with LPS for 48 h, treated with EdU for 2 h, and analyzed by flow cytometry for cell cycle progression. The error bars represent the standard errors of the means (SEMs). ns, not significant; *p < 0.05, **p < 0.01 and ***p < 0.001. The data shown are representative of three independent experiments

We wondered whether reduced GCB formation, plasma cell differentiation, and antibody production resulting from B-cell-specific deletion of eIF4A1 and eIF4A2 were caused by impaired B-cell activation and proliferation. B cells were purified from *Eif4a1*^*fl/fl*^*;CD19Cre* and *Eif4a2*^*fl/fl*^*;CD19Cre* mice, stimulated with LPS, and examined for activation and proliferation. As shown in Fig. 4C, F, the absence of either eIF4A1 or eIF4A2 had no obvious effect on B-cell activation, as indicated by the normal upregulation of activation markers CD83, CD86, and CD69. Instead, it severely impaired B-cell proliferation by blocking the G1/S transition during cell cycle progression (Fig. 4D, E, G, H). Taken together, our findings show that eIF4A1 and eIF4A2 are dispensable for activation of B cells, but essential for their cell cycle progression and proliferation.

### eIF4A2 controls the biogenesis of the 40S ribosome subunit

**Fig. 5:**
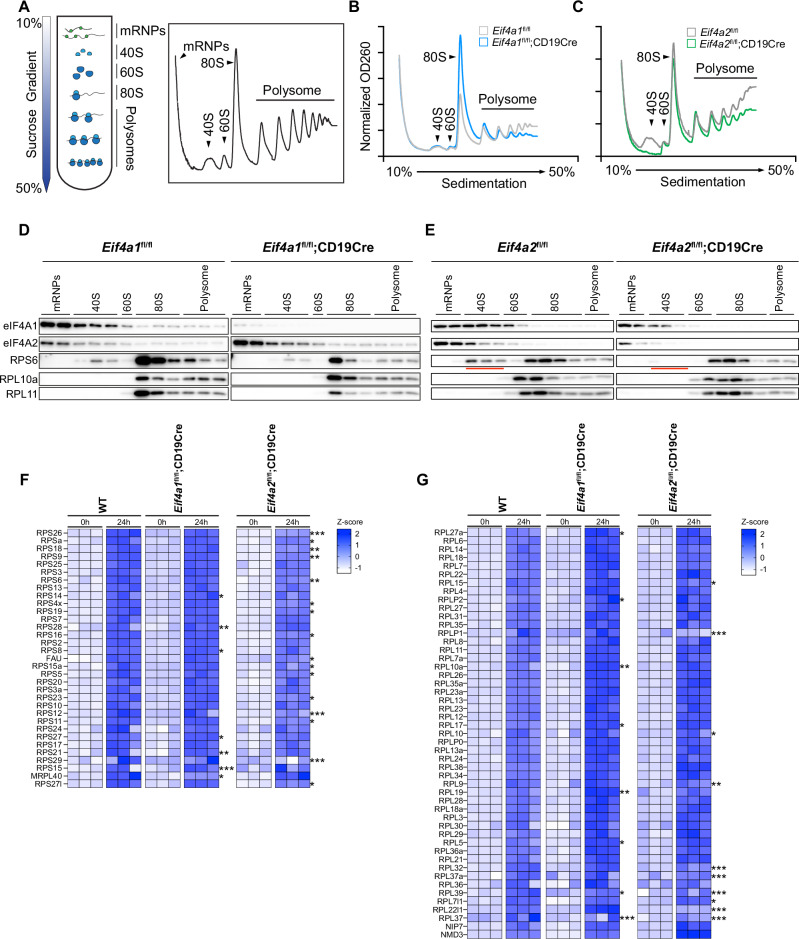
**eIF4A2 is required for 40S ribosome biogenesis**. **A** Outline of polysome profiling experiments. **B, C** Polysome profiling graphs of *Eif4a1*^fl/fl^, *Eif4a1*^fl/fl^;CD19Cre **B**, *Eif4a2*^fl/fl^, *Eif4a2*^fl/fl^;CD19Cre **C** B cells stimulated with LPS for 24 h. **D, E** Immunoblot analysis of eIF4A1, eIF4A2, and ribosomal proteins in the indicated sucrose gradient fractions of B-cell lysates. Red underlines indicate the absence of RPS6 protein in the 40S fractions of *Eif4a2*-deficient B cells **E**. **F, G** Heatmap of RPS **F** and RPL **G** proteins in WT (C57BL/6 J), *Eif4a1*^fl/fl^;CD19Cre and *Eif4a2*^fl/fl^;CD19Cre B cells stimulated with LPS for the indicated durations. Two-way ANOVA was performed. The asterisks on the right side of each panel indicate the differences between WT and *Eif4a1*- or *Eif4a2*-deficient B cells at 24 hours after LPS stimulation **F, G.** *p < 0.05, **p < 0.01 and ***p < 0.001. The data shown are representative of three independent experiments. Each column represents an individual biological sample

Previous studies have shown that eIF4A1 and eIF4A2 are components of the eIF4F complex and play critical roles in translation initiation by unwinding secondary structures in the 5’UTRs of mRNAs. We therefore examined the impact of their absence on global translation in B cells. Naïve B cells from *Eif4a1*^*fl/fl*^*;CD19Cre and Eif4a2*^*fl/fl*^*;CD19Cre* mice were activated with LPS for 24 hours. The cytoplasmic contents were harvested and separated in a sucrose density gradient (Fig. 5A**)**. As shown in Fig. 5B, there was an overall reduction in heavy polysome fractions accompanied by a significant accumulation of 80S monosome fractions in *Eif4a1*-deficient B cells, indicating a decrease in the global translation rate. Strikingly, *Eif4a2* deficiency resulted in a drastic reduction in the 40S fraction and an overall reduction in the heavy polysome fractions, while the 80S monosome fraction was not much affected (Fig. 5C). The polysome profiling results were further corroborated by immunoblot analysis of ribosomal proteins in polysome fractions. As shown in Fig. 5D, E, while *Eif4a1*-deficient B cells exhibited an overall reduction of ribosomal proteins in 80S and polysome fractions, the small ribosome subunit protein RPS6 was almost absent from the 40S fractions of *Eif4a2*-deficient B cells. This, together with the drastic reduction in 40S fractions in the polysome profiles of *Eif4a2*-deficient B cells (Fig. 5C), prompted us to hypothesize that eIF4A2 controls RPS protein expression and biogenesis of the 40S subunit of ribosome. 40S ribosome contains 18S rRNA and 33 ribosomal proteins (RPs). Eukaryotic ribosome assembly starts with the transcription of a polycistronic 47S rRNA precursor in the nucleolus. Emerging rRNA stretches are quickly bound by early assembly RPs and ribosome biogenesis factors (RBFs), giving rise to the 90S precursor particle, which is subsequently separated into pre-40S and pre-60S particles through a critical endonucleolytic pre-rRNA cleavage event. Both particles undergo numerous maturation steps, nuclear export, and final assembly in the cytoplasm, giving rise to mature and translationally competent 40S and 60S ribosome subunits [24]. We quantified RPS and RPL proteins in *Eif4a1- and Eif4a2*-deficient B cells before and after LPS stimulation by label-free quantitative mass spectrometry (LFQ-MS). As shown in Fig. 5F, G, LPS stimulation substantially elevated the expression of RPS and RPL proteins. While *Eif4a1* deficiency had marginal effect on Eif4a2 upregulation, *Eif4a2* deficiency significantly impaired upregulation of RPS proteins and, to a lesser degree, RPL proteins. Taken together, these data suggest that eIF4A1 controls global translation, while eIF4A2 regulates LPS-induced upregulation of RPS protein expression and biogenesis of the 40S ribosome subunit.

### eIF4A2 is required for 18S rRNA maturation

**Fig. 6:**
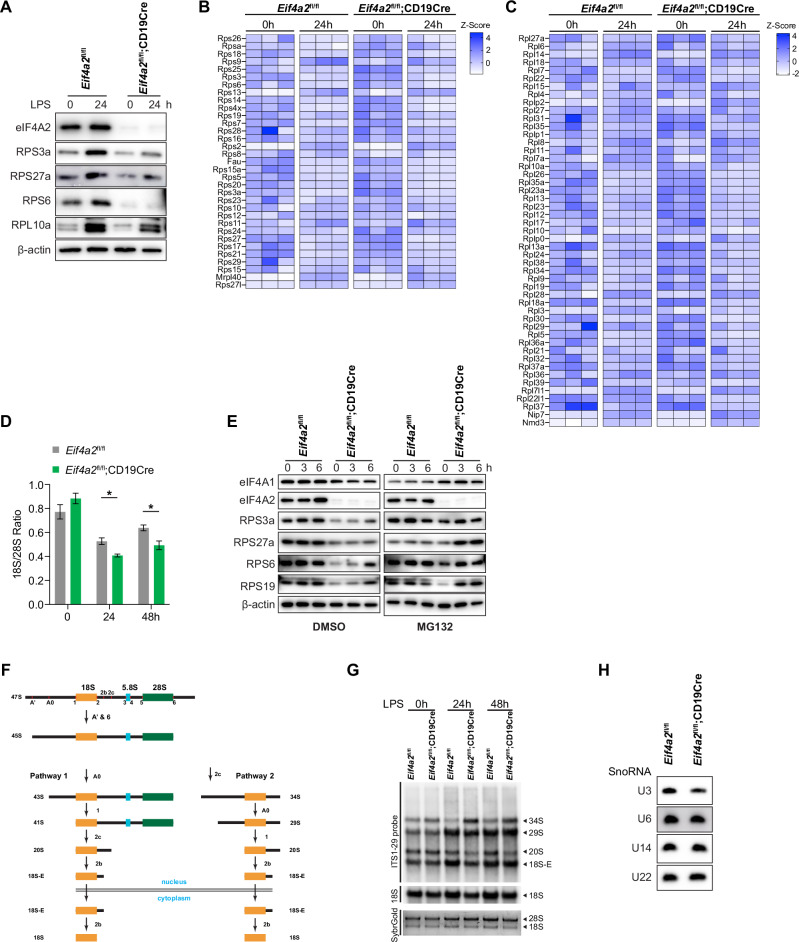
**eIF4A2 is required for 18S rRNA maturation**. **A** Immunoblot analysis of eIF4A2 and ribosomal proteins in *Eif4a2*^fl/fl^ and *Eif4a2*^fl/fl^;CD19Cre B cells stimulated with LPS for the indicated amounts of time. **B, C** Heatmap of the mRNA levels of the RPS **B** and RPL **C** genes determined by RNA-seq analysis of *Eif4a2*^fl/fl^ and *Eif4a2*^fl/fl^;CD19Cre B cells stimulated with LPS for the indicated amounts of time. **D** The 18S/28S rRNA ratio was quantified via the Agilent 5400 Fragment Analyzer System to measure the amounts of 18S and 28S rRNA in total RNA from *Eif4a2*^fl/fl^ and *Eif4a2*^fl/fl^;CD19Cre B cells stimulated with LPS for the indicated amounts of time. **E** *Eif4a2*^fl/fl^ and *Eif4a2*^fl/fl^;CD19Cre B cells were stimulated with LPS for 12 hours, treated with MG132 or DMSO for the indicated amounts of time, and analyzed by immunoblotting. **F** Scheme of 18S rRNA maturation. **G, H** Northern blot analysis of total RNA from B cells stimulated with LPS for 24 hours using ITS-29 **G**, U3, U6, U14, and U22 probes **H**. The error bars represent the standard errors of the means (SEMs). ns, not significant; *p < 0.05, **p < 0.01 and ***p < 0.001. The data shown are representative of three independent experiments

Lymphocyte activation is accompanied by a drastic increase in the number of ribosomes, translational output, and protein synthesis to sustain cellular growth and proliferation. In mammalian cells, ribosome biogenesis is controlled by two main mechanisms: rRNA synthesis and ribosomal protein expression. Immunoblot analysis of individual ribosomal proteins confirmed that the LPS-induced upregulation of RPS proteins was impaired in *Eif4a2*-deficient B cells (Figs. 5F, G, 6A). RNAseq analysis of the transcriptomes of *Eif4a*2^fl/fl^ and *Eif4a*2^fl/fl^;CD19Cre B cells before and after LPS stimulation revealed little difference in the mRNA expression levels of RPS and RPL proteins between time points and genotypes (Fig. 6B, C), suggesting that eIF4A2 regulates RPS protein expression mainly through post-transcriptional mechanisms.

Previous studies have shown that ribosomal protein expression is mainly controlled by the rate of rRNA synthesis [25]. Ribosomal proteins are often synthesized at high levels beyond that required for ribosome subunit production. This is balanced by continual degradation of ribosomal proteins not assembled with rRNA. Indeed, the 18S/28S rRNA ratio in LPS-activated *Eif4a*2^fl/fl^;CD19Cre B cells was significantly less than that in *Eif4a*2^fl/fl^ B cells (Fig. 6D), suggesting compromised biogenesis of 18S rRNA. In addition, treatment of LPS-stimulated B cells with MG132, a proteasome inhibitor, substantially restored the expression of RPS proteins in *Eif4a2*-deficient B cells (Fig. 6E). Taken together, these results show that impaired upregulation of RPS proteins in LPS-activated *Eif4a2*-deficient B cells is mainly caused by compromised biogenesis of 18S rRNA and subsequent degradation of RPS proteins not assembled with 18S rRNA.

This finding prompted us to examine the maturation of 18S rRNA, the RNA component of the 40S ribosome subunit, in *eIF4A2*-deficient B cells. 18S rRNA is generated from the 47S rRNA precursor through a complex series of endonucleolytic and exonucleolytic cleavage steps (Fig. 6F) [26]. Northern blot analysis of 47S rRNA processing intermediates showed significant accumulation of 34S rRNA, reduction of 20S and 18S-E, and decrease of mature 18S, but not 28S, rRNA (Fig. 6G and Supplementary Fig. 4). We investigated the expression of several small nucleolar RNAs that play critical roles in 18S rRNA biogenesis [27–30]. As shown in Fig. 6H, U3 expression was reduced in eIF4A2-deficient B cells, whereas the levels of U6, U14, and U22 were comparable between *Eif4a*2^fl/fl^ and *Eif4a*2^fl/fl^;CD19Cre B cells. Taken together, these findings show that eIF4A2 controls 40S ribosome subunit biogenesis by regulating 18S rRNA maturation.

### eIF4A1 controls the cell cycle through translational regulation of Gins4 and other genes

**Fig. 7:**
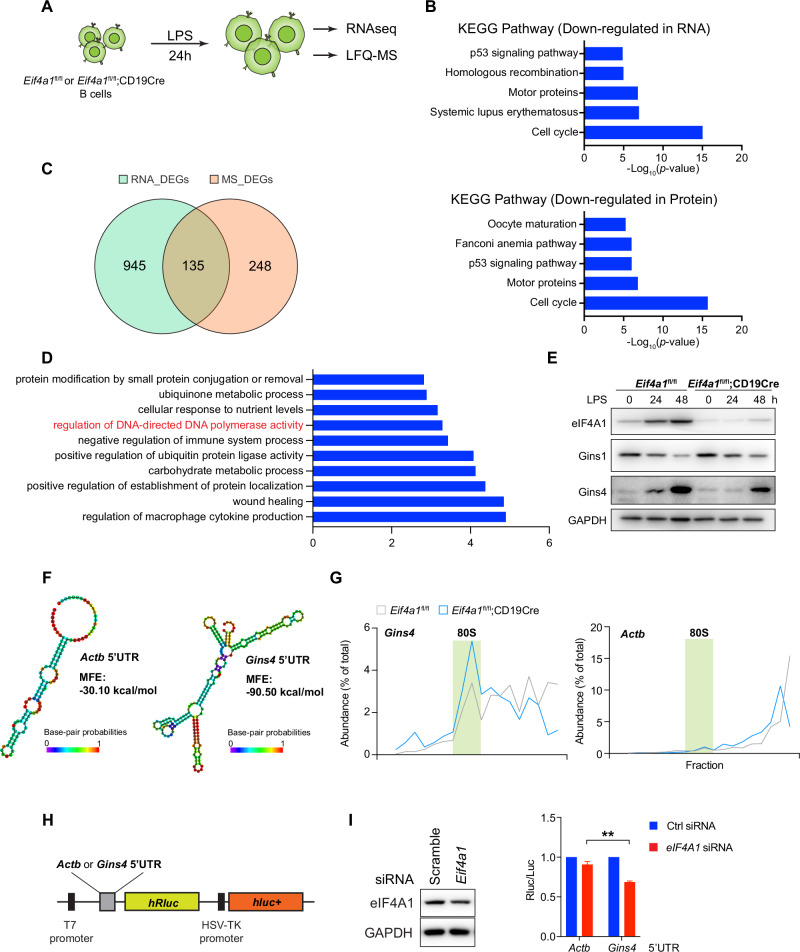
**eIF4A1 controls the translation of** ***Gins4*** **via 5’UTR**. **A** Experimental outline of the RNAseq and LFQ-MS experiments. **B** KEGG pathway enrichment of downregulated proteins identified by LFQ-MS and downregulated mRNAs quantified by RNA-seq. **C** Venn diagram analysis of differentially expressed genes (DEGs) identified via RNA-seq (RNA-DEGs) and LFQ-MS (MS-DEGs). Cutoff, absolute Log2FoldChange greater than 0.5. **D** A total of 248 genes from **C** with only altered protein expression were analyzed via Metascape for GO enrichment, and the top ten terms were plotted. **E** Immunoblot analysis of eIF4A1, Gins1, and Gins4 in *Eif4a1*^fl/fl^ and *Eif4a1*^fl/fl^;CD19Cre B cells stimulated with LPS for the indicated amounts of time. **F** RNA structures of *Gins4* and *Actb* 5’UTRs were predicted via RNAfold. MFE, minimum free energy. **G** Distribution of *Actb* and *Gins4* mRNAs in the sucrose gradient fractions of *Eif4a1*^fl/fl^ and *Eif4a1*^fl/fl^;CD19Cre B cells stimulated with LPS for 24 hours. **H** Graphical outline of the luciferase reporter gene. **I** Immunoblot analysis of eIF4A1 protein expression in HEK293T cells transfected with scramble or *Eif4a1* siRNA (left panel). Luciferase assay of the reporter gene harboring the *Actb* or *Gin4* 5’UTR **H** in HEK293T cells transfected with scramble or *Eif4a1* siRNA (right panel). The error bars represent the standard errors of the means (SEMs). ns, not significant; *p < 0.05, **p < 0.01 and ***p < 0.001. The data shown are representative of three independent experiments

We next investigated the molecular mechanisms underlying eIF4A1 control of the cell cycle. B cells from *Eif4a*1^fl/fl^ and *Eif4a*1^fl/fl^;CD19Cre mice were stimulated with LPS for 24 hours and analyzed by label-free quantitative mass spectrometry (LFQ-MS) and RNA-seq to elucidate the impact of eIF4A1-deficiency on the proteome and transcriptome, respectively (Fig. 7A). While RNAseq analysis identified 533 up-regulated and 547 down-regulated genes, mass spectrometry analysis revealed 163 up-regulated and 220 down-regulated proteins. Genes with protein or RNA levels downregulated in *Eif4a1*-deficient B cells were analyzed for KEGG pathway enrichment. Consistent with the critical role of eIF4A1 in controlling cellular proliferation (Fig. 4D, E), the cell cycle was the most significantly enriched molecular pathway among the genes downregulated in either protein or RNA level (Fig. 7B). Previous studies have demonstrated critical roles of eIF4A1 in controlling translation initiation [31]. *Eif4a1*-deficient B cells exhibited a decrease in the global translation rate (Fig. 5B). We reasoned that direct target genes of eIF4A1 should exhibit alterations in protein, but not mRNA, levels in *Eif4a1*-deficient B cells. We therefore compared the differentially expressed genes in RNA levels (RNA-DEGs) with the differentially expressed genes in protein levels (MS-DEGs) and identified 248 DEGs mainly altered at the protein level (Fig. 7C). Metascape analysis of these genes identified a gene set of “regulation of DNA-directed DNA polymerase activity” (Fig. 7D), which includes Gins1 and Gins4, proteins of the Cdc45-MCM-GINS (CMG) complex. The CMG complex functions in the DNA melting and unwinding steps as a component of replisome during DNA replication in mammalian cells [32]. We speculate that these Gins proteins are the main mediators of eIF4A1 control of the cell cycle. Immunoblot analysis showed that the Gins4 protein was significantly induced upon LPS stimulation of *Eif4a1*^fl/fl^ B cells and that this induction was much less in the absence of eIF4A1 (Fig. 7E). eIF4A1 promotes cap-dependent translation initiation by unwinding secondary structures in the 5’UTR of mRNA [1]. Indeed, the 5’UTR of *Gins4* mRNA harbors highly complicated secondary structures when compared to *Actb* mRNA. Moreover, the minimum free energy of *Gins4* 5’UTR secondary structures is −90.50 kcal/mol, which is three-fold lower than that of *Actb* 5’UTR (Fig. 7F), suggesting a critical role of eIF4A1 in unwinding secondary structures of the *Gins4* 5’UTR to promote translation initiation of this mRNA. We examined the distribution of *Actb* and *Gins4* mRNAs in the sucrose gradient (Fig. 7G). While the distribution of *Actb* mRNA showed a slight shift from heavy to light polysomes in *Eif4a1*-deficient B cells, *Gins4* mRNA showed a much greater shift toward 80S monosome and light polysome fractions, indicating a strong dependency of *Gins4* mRNA on eIF4A1 for translation initiation.

**Fig. 8:**
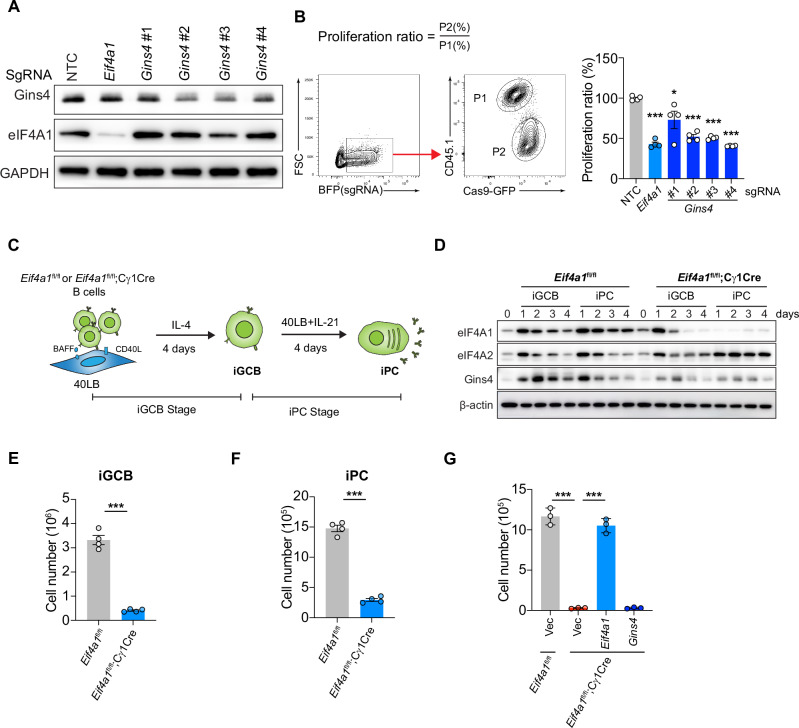
**eIF4A1 exerts its functions in B cells through Gins4 and other genes**. **A** Immunoblot analysis of Gins4 and eIF4A1 protein expression in LPS-stimulated Cas9-GFP B cells transduced with retroviruses encoding nontargeting control (NTC), *Eif4a1* or *Gins4* sgRNA. The retroviral vector encodes blue fluorescent protein (BFP), and transduced cells are BFP positive. **B** Cas9-GFP and wild-type B cells (CD45.1 + ) were mixed at a 1:1 ratio, stimulated with LPS for 24 hours, transduced with retroviruses encoding nontargeting control (NTC), *Eif4a1* or *Gins4* sgRNA, and analyzed by flow cytometry at 72 hours post retroviral transduction. The proliferation ratio was calculated as indicated. **C** Scheme of iGCB culture. **D** Immunoblot analysis of eIF4A1, eIF4A2 and Gins4 proteins in *Eif4a1*^fl/fl^ and *Eif4a1*^fl/fl^;Cγ1Cre B cells cultured in the iGCB system at the indicated time points. **E, F** Numbers of *Eif4a1*^fl/fl^ and *Eif4a1*^fl/fl^;Cγ1Cre iGCB **E** and iPC **F** cells at the end of the indicated stages of culture. **G** *Eif4a1*^fl/fl^ and *Eif4a1*^fl/fl^;Cγ1Cre B cells were cultured in the iGCB system and transduced with retroviruses encoding *Eif4a1* or *Gins4* at iGCB day 2.5. Transduced cells (GFP + ) were sorted at iGCB day 4 and replated on 40LB cells in the presence of IL-21 for iPC differentiation. The numbers of cells at the end of iPC culture were plotted. The data shown are representative of three independent experiments

We further examined this in a reporter assay. The *Gins4* and *Atcb* 5’UTRs were cloned into the *psi*Check2 reporter plasmid and placed in the 5’UTR of the humanized Renilla luciferase (hRLuc) gene (Fig. 7H). As shown in Fig. 7I, siRNA-mediated knockdown of eIF4A1 significantly downregulated the hRluc activity of the reporter gene containing the *Gins4* 5’UTR. Furthermore, CRISPR/Cas9-mediated deletion of *Gins4* and *Eif4a1* caused a similar impairment in B cell proliferation (Fig. 8A, B), suggesting that *Gins4* is a major mediator of eIF4A1 control of cell cycle.

We then asked whether ectopic expression of *Gins4* is able to restore cellular proliferation of *Eif4a1-*deficient B cells. As *Eif4a*1^fl/fl^;CD19Cre B cells show impaired cellular proliferation and cannot be transduced by retroviruses, we bred *Eif4a*1^fl/fl^ mice with Cγ1Cre mice, in which the expression of Cre is induced by transcription of the Igγ constant region gene segment (Cγ1) [33]. B cells from *Eif4a*1^fl/fl^;Cγ1Cre and *Eif4a*1^fl/fl^ mice were analyzed in an in vitro culture system mimicking B-cell differentiation into plasma cells. In this system, naïve B cells were cultured on top of BALB/c 3T3 cells stably expressing CD40L and Baff (termed 40LB cells). In the presence of IL-4, naïve B cells acquire a GCB cell phenotype (Fas^+^GL-7^+^Bcl6^+^, termed iGCB cells) after 4 days of culture. Those iGCB cells can further differentiate into plasma cells (termed iPC cells) after 4 additional days of culture in 40LB cells in the presence of IL-21 (Fig. 8C) [34]. *Eif4a*1^fl/fl^;Cγ1Cre B cells showed significant reduction in eIF4A1 protein expression on day 2 and complete depletion on day 3 of the iGCB stage of culture (Fig. 8D). Consistent with previous observations in LPS-stimulated B cells (Fig. 7E), *Eif4a1*-deficient B cells expressed much lower amounts of the Gins4 protein than *Eif4a*1^fl/fl^ B cells (Fig. 8D). The numbers of *Eif4a1*-deficient B cells at the end of the iGCB and iPC stages of culture were also much reduced (Fig. 8E, F). We then transduced *Eif4a*1^fl/fl^;Cγ1Cre B cells with retroviruses encoding eIF4A1 or Gins4 on day 2.5 of the iGCB stage. As shown in Fig. 8G, retroviral expression of eIF4A1 fully restored the number of *Eif4a*1^fl/fl^;Cγ1Cre B cells at the end of iPC culture, while retroviral expression of Gins4 had no obvious effect. Therefore, eIF4A1 likely controls cellular proliferation through multiple target genes, with Gins4 as one of them.

## Discussion

We took a mouse genetic approach to study the functions of eIF4A1 and eIF4A2 during B-cell development and humoral immune responses. Our results showed that they play critical but differential roles in the B-cell lineage. While *Mb1Cre*-mediated deletion of eIF4A1 right after B cell lineage commitment led to impaired development at Hardy fractions (Frs.) B and C, deletion of eIF4A2 caused developmental block at Fr.D. During T-cell-dependent antibody responses, both eIF4A1 and eIF4A2 are required for germinal center B cell formation, plasma cell differentiation, and antibody production. During T-cell-independent antibody responses, eIF4A1 is required only for the TI-1 antibody response, whereas eIF4A2 is required for both the TI-1 and TI-2 antibody responses. At the cellular level, both eIF4A1 and eIF4A2 control the activation-induced cell cycle progression and proliferation of B cells. In spite of seemingly similar cellular functions, eIF4A1 and eIF4A2 exert drastically different molecular functions. While eIF4A1 regulates the global translational rate, eIF4A2 mainly promotes 18S rRNA maturation and biogenesis of the 40S ribosome subunit.

The expression patterns of eIF4A1 and eIF4A2 are consistent with their distinct molecular functions. Naïve B cells express very little eIF4A1 protein. Upon activation by various stimuli, eIF4A1 is strongly induced and maintained at high levels to support global protein synthesis. This is consistent with its role in the eIF4F complex to unwind secondary structures in the 5’UTR of mRNA to promote 5’UTR scanning by the 43S preinitiation complex [1]. In contrast, eIF4A2 protein expression is transiently induced upon B cell activation and decreases after the first 24 hours. This is suitable for its role in promoting ribosome biogenesis. Recent studies have shown that naïve lymphocytes assume a poised state at the translational level, expressing substantial amounts of mRNAs encoding ribosomal proteins and translation initiation and elongation factors. In those naïve cells, there is a large pool of idling ribosomes whose translational output is maintained at a low level. Upon activation, translational output of pre-existing ribosomes is increased by 4-fold in the first 6 hours. However, this pool of pre-existing ribosomes is not sufficient to sustain cell growth and proliferation. In the first 24 hours of activation, the number of ribosomes per cell would increase by 6-fold. This, together with the elevated translational output of individual ribosomes, leads to more than 20-fold increase in the total output of newly synthesized proteins. The drastic increase in protein synthesis is essential for the growth and proliferation of activated lymphocytes [35, 36]. The rapid induction of eIF4A2 is critical for the production of new ribosomes in the first 24 hours of lymphocyte activation. As ribosomes are relatively stable and have long half-life, there is no need to maintain eIF4A2 at high levels after the number of ribosomes reaches a level sufficient to sustain cellular growth and proliferation. This may explain the decrease in eIF4A2 protein levels after the first 24 hours of lymphocyte activation.

Ribosome biogenesis is controlled by two main mechanisms: rRNA synthesis and ribosomal protein expression. Previous studies showed that ribosomal protein expression is mainly controlled by the rate of rRNA synthesis [25]. Our study showed that in the absence of eIF4A2, 18S rRNA maturation is compromised in activated B cells. This led to the degradation of RPS proteins not assembled with 18S rRNA and much reduced the number of 40S ribosome subunits. 18S rRNA is generated from the 47S rRNA precursor through a complex series of endonucleolytic and exonucleolytic cleavage steps [26]. Our study showed that eIF4A2 deletion impaired multiple steps of 47S rRNA processing, resulting in reduced amounts of 18S rRNA. This role of eIF4A2 in regulating 18S rRNA maturation was previously unknown. Past studies showed that the nascent primary transcript of 47S rRNA associates co-transcriptionlly with some ribosomal proteins, numerous pre-ribosomal factors (PRFs), and small nucleolar ribonucleoprotein particles (snoRNPs) to form a series of large RNPs, in which pre-rRNA folding, modification, processing, and ribosomal protein assembly take place [26]. Among the small nucleolar RNAs that play critical roles in 18S rRNA biogenesis [27–30], we found that U3 expression was reduced, while the expression of U6, U14, and U22 remained unaltered in *eIF4A2*-deficient B cells. Future investigations are warranted to elucidate the molecular mechanisms underlying eIF4A2 control of 18S rRNA biogenesis.

## Methods and materials

### Mice

*Eif4A1*^fl/fl^ and *Eif4a2*^fl/fl^ mice on the C57BL/7 J background were generated by Xiamen University Laboratory Animal Center with the method of haploid embryonic stem cells [37]. Briefly, ribonucleoproteins (RNPs) consisting of the Cas9 protein, sgRNA pairs targeting *Eif4a1* or *Eif4a2*, and *lox*P-flanked DNA templates were injected into haploid embryonic stem cells. Haploid embryonic stem cells carrying the desired genome modification were identified by PCR and injected into oocytes, followed by in vitro culture and embryo transfer into pseudopregnant female mice. C57BL/7 J (Jax stock: 000664), Mb1Cre (Jax stock: 020505), CD19Cre (Jax stock: 006785) and Cγ1Cre (Jax stock: 010611) mice have been previously reported. Mice were analyzed at the age of 6–8 weeks for B-cell development. 8-12 week-old age- and gender-matched mice were used for immunization and B cell isolation. All the mice were housed under specific pathogen-free (SPF) conditions. All animal experiments were approved by Animal Care and Use Committee of Xiamen University.

### Cell lines

293 T cells were cultured with complete DMEM (Gibco) medium supplemented with 10% FBS (ExCell), 1 × NEAA (Gibco) and 100 U/mL penicillin and streptomycin (Gibco). 40LB cells were previously reported [34], maintained in complete DMEM medium, irradiated with 120 Gy X-ray, harvested and frozen at −80°C with CELLSAVING (NCM Biotech, C40100), and replated before use.

### Immunization and ELISA

For analysis of GCB and plasma cells, mice were immunized intraperitoneally with 100 μg OVA precipitated with alum and 10 μg LPS (OVA/Alum/LPS). Splenocytes were harvested for flow cytometry analysis 7.5 days post immunization. For NP-LPS immunization, 50 μg of NP-LPS (Biosearch Technologies; N-5065-5) was intravenously injected and the serum was collected at indicated time points. For antigen-specific antibody analysis, mice were immunized intraperitoneally with 10 μg of NP-OVA (Biosearch Technologies; N-5051-100) precipitated with alum (NP-OVA/Alum), and the serum was collected at indicated time points. Concentrations of NP-specific antibodies in the serum were determined by ELISA as previously reported [38]. Briefly, microplates (Thermo Fisher, 439454) were coated with 10 μg/mL NP_30_-BSA (for total IgG1 or IgM measurement) or NP_5_-BSA (for high-affinity IgG1 measurement). Nonspecific binding was blocked with PBS supplemented with 0.5% BSA (PBSA). Serum samples were serially diluted in PBSA and incubated in blocked plates at room temperature for 2 hours. The plates were incubated with biotin-conjugated anti-IgM (Southern Biotech, 1020--08) or anti-IgG1 (Southern Biotech, 1070-08) and with streptavidin-alkaline phosphatase (Roche) for 1 hours, followed by incubation with alkaline phosphatase substrate solution containing 4-nitro-phenyl phosphate (Sigma) for color development. Plates were quantified on a VERSAmax microplate reader (Molecular Devices).

### Primary B-cell culture

Naïve B cells from the spleen were purified by negative selection with BeaverBeads Streptavidin (1 μM) (BEAVER, 22307) and biotin-conjugated antibodies against CD5, CD9, CD43, CD93 and Ter-119. B cells were cultured with B-cell medium, which is RMPI-1640 supplemented with 10% FBS (HyClone), 10 mM HEPES, 50 μM β-mercaptoethanol (Sigma), 100 U/mL penicillin and streptomycin (Gibco). For in vitro B-cell stimulation, naïve B cells were suspended in B cell medium at a density of 1 × 10^6^ cells/mL and stimulated with 5 μg/ml LPS (Sigma). For iGCB culture, naïve B cells were cultured for the 1st stage with irradiated 40LB plus IL-4 (1 ng/mL, Novoprotein, CK74) for 4 days, followed by 2nd stage culture with irradiated 40LB cells plus IL-21 (10 ng/mL, Novoprotein, CK10) for another 4 days.

### FACS analysis

Single cell suspensions were freshly prepared from the spleen, bone marrow and peripheral lymph nodes followed by red blood cell lysis. A total of 1 ~ 2×10^6^ cells were stained for surface markers in FACS buffer (PBS with 0.5% BSA and 0.05% NaN_3_) at 4°C for 30 min, washed and resuspended in FACS buffer. Cell cycle staining was performed following the instructions of the BeyoClick™ EdU Cell Proliferation Kit with an Alexa Fluor 647 Kit (Beyotime; C0081S). Briefly, naïve B cells were stimulated with LPS (5 μg/mL) for 48 hours. EdU (10 μM) was added 2 hours prior to cell harvest. Cells were collected by centrifugation, washed three times with PBS, fixed with BD Fix/Perm solution (Cat No: 51--2090KZ) for 30 min at room temperature (RT), and washed three times with PBS. EdU-incorporated cells were incubated in click reaction cocktail for 30 min in the dark at room temperature. After click reaction, cells were washed with PBS,resuspended in PBS containing Hoechst 33342, followed by FACS analysis of the cell cycle. Flow cytometry data were acquired on a Fortessa or LSRFortessa X-20 (BD Biosciences), or NovoCyte flow cytometer (ACEA Biosciences, Agilent) and analyzed with FlowJo software 10 (Treestar).

### Polysome profiling

Polysome profiling was performed following a protocol previously reported by our laboratory [39]. Briefly, naïve B cells were stimulated with LPS (5 μg/mL) for 24 hours. Activated B cells were harvested after 15 min treatment with 100 μg/mL cyclohexamide (CHX), followed by washing with 10 mL hypotonic buffer (1.5 mL of KCl, 10 mM MgCl2, 5 mM Tris-HCl (pH 7.4), and 100 μg/mL CHX). Cells were pelleted, resuspended gently in 300 μL of hypotonic buffer, followed by addition of an equal volume of hypotonic lysis buffer (2% sodium deoxycholate, 2% Triton X-100, 2.5 mM DTT, 10 units of RNase inhibitor/mL, and 100 μg/mL CHX) and incubation on ice for 20 min. Supernatants were collected as cytosolic fractions after centrifugation at 13,000 × g for 10 min. Cytosolic fractions were loaded onto a 10–50% sucrose gradient containing RNase inhibitor and centrifuged at 38,000 rpm for 2 hours using a Beckman SW41 rotor. The sucrose gradient was separated into 20 fractions from the top, and absorbance at 260 nm was monitored continuously by a Piston gradient fractionator (Biocomp, Canada).

### Retroviral transduction

293 T cells were plated at 1.5 × 10^6^ cells per well in 6-well plates one day before transfection to reach ~80% confluency on the day of transfection. Cell culture medium was replaced with fresh complete DMEM 1 ~ 2 hours prior to transfection. Cells in each well were transfected with 3 μg of retroviral vector and 1 μg of packaging plasmid (pCl-Eco, Addgene #12371) by the calcium phosphate method. 36 ~ 48 hours post-transfection, the supernatant containing viruses were collected and filtered with 0.45 μM filters. For iGCB culture, naïve B cells were purified, plated in 6-well plates, and transduced with retroviruses on day 2 of culture by spinoculation. For LPS stimulation, B cells were stimulated with LPS for 24 hours, followed by addition of retroviruses and spinoculation.

### Immunoblotting

Cell pellets were lysed with RIPA buffer and cleared by centrifugation at 14,000 rpm, 4°C for 15 min. Supernatants were collected, resolved on SDS‒PAGE gels, and transferred to PVDF membranes (Millipore, IPVH00010). The membrane was incubated overnight at 4°C with primary antibodies diluted in 1× Tris-buffered saline (TBS) (10 mM Tris-HCl, pH 8.0, and 150 mM NaCl) with 5% (wt/vol) BSA or nonfat milk, washed 3 times in TBS buffer with 0.5% Tween 20, and incubated with horseradish peroxidase (HRP)-conjugated goat anti-rabbit or goat anti-mouse antibodies in TBS at room temperature for 1 hour. The membrane was then washed 3 times in TBS buffer with 0.1% Tween 20. Protein bands were visualized with ECL Select Western Blotting Detection Reagent (GE Healthcare) or Immobilon Western Chemiluminescent HRP Substrate (Merck Millipore) following the manufacturer’s instructions (GE Healthcare). Images were acquired with Amersham Imager 600 (GE Healthcare).

### Northern blot

Total RNA from B cells was extracted by TRIzol following the manufacturer’s instructions and quantified with a NanoDrop spectrophotometer. 5–10 μg RNA was separated by electrophoresis on 1.2% denaturing agarose–formaldehyde gels (for rRNA detection) or 10% denaturing urea polyacrylamide gels (for snoRNA detection), transferred to Immobilon-Ny^+^ membranes, followed by UV crosslinking. Membranes were incubated with biotinylated DNA probe, and then with horseradish peroxidase (HRP)-conjugated streptavidin. Bands were visualized by chemiluminescence methods. For rRNA separation, we optimized the method based on the basis of previously published work [40]. The specific probes used were as follows: ITS1-29 [41]: agccgccgctcctccacagtctcccgtt; 18S [42]: GGCCGTGCGTACTTAGACAT; U3: TCTTCCTTGTGGTCTTGGGTGCTC; U14: AGCACTT- CTGGTGGAAACTACGAATGG; U22: CAGTGGGTTCACCTTTTCAGGCTCT; and U6: tgtgctgaggtaagcac.

### RNAseq and data analysis

B cells from three *Eif4a*1^fl/fl^;CD19Cre, *Eif4a*1^fl/fl^, *Eif4a*2^fl/fl^;CD19Cre, and *Eif4a*2^fl/fl^ mice were activated with LPS (5 μg/mL) for 24 hours, harvested, and washed with cold PBS for three times. Total RNA from activated B cells was isolated by RNeasy Kit (QIAGEN) following the manufacturer’s instructions. After total RNA isolation, sequencing libraries were analyzed with an NEBNext® UltraTM RNA Library Prep Kit from Illumina® (NEB, USA) following the manufacturer’s instructions. The clustering of the index-coded samples was performed on a cBot Cluster Generation System via the TruSeq PE Cluster Kit v3-cBot-HS (Illumina) following the manufacturer’s instructions. After cluster generation, the library preparations were used for sequencing  on an Illumina NovaSeq platform (Novogene, Beijing). The raw fastq files were processed with Trimmomatic to remove adapter sequences and low-quality reads. Filtered reads were aligned to mouse reference genome (mm39) via STAR (version 2.5.2b) with default parameters. Raw counts were generated by featureCounts (Version 1.6.2), normalized and analyzed by DESeq2 in R. Differential expression analysis was performed with cutoffs of *p* < 0.05 and absolute log2FoldChange > 1. Gene Ontology (GO) enrichment of differentially expressed genes was performed with clusterProfiler R package with corrected *P* value < 0.05.

### Label-free quantitative mass spectrometry (LFQ-MS)

The cells were collected by centrifugation at 200 × g for 10 min, followed by washing with ice-cold PBS for three times. Cells were lysed with 1% SDS/10 mM TCEP/40 mM CAA/100 mM Tris-HCl followed by incubation at 95°C for 10 min. Cell lysates were sonicated. The protein concentration was measured BCA kit (Thermo Fisher Scientific) followed by SCASP [43]. HP-β-cyclodextrin was added to samples to trap SDS (30 μl 250 mM HP-β-cyclodextrin into 100 μl SDS samples). Subsequently, trypsin was subsequently added at a protein:trypsin ratio of 50:1. Digestion was performed at 37°C overnight. Peptides were cleaned up using SDB-RPS StageTips. Peptide samples were resolved in 2% ACN/0.1FA for LC‒MS analysis. The LC used was an EASY-nLC 1200 system (Thermo Fisher Scientific, San Jose, CA) harboring a home-pulled emitter integrative 75 µm by 35 cm C18 column packed with 1.8 µm 120 Å C18 material (Welch, Shanghai, China). A 135-min LC separation was configured on the basis of the mixture of buffer (0.1% formic acid in H_2_O) and buffer B (80% acetonitrile containing 0.1% formic acid). Buffer B was made to increase from 4 to 34% in 120 min, surge to 100% in 3 min, and keep at 100% for 8 min. The LC‒MS flow rate was kept at 300 nl/min. The Orbitrap Fusion Lumos Tribrid mass spectrometer (Thermo Fisher Scientific) instrument coupled to a nanoelectrospray ion source (NanoFlex, Thermo Fisher Scientific) was used. All the DIA-MS methods consisted of 1 MS1 scan and 40 MS2 scans of variable windows by quadrupole isolation. DIA files (raw) were loaded into DIA-NN (v1.8) [44]. Mouse FASTA files downloaded from UniProt/Swissprot were added. “FASTA digest for library-free search” and “Deep learning-based spectra, RTs and IMs prediction” were enabled. “Generate spectral library” was enabled. “Protein inference” was set to “protein name” (from FASTA). Other parameters were left as defaults (Supplementary Table 1).

### Statistical analysis

All the data are presented as the means ± SEMs. Statistical analysis was performed with GraphPad Prism 9. P-values  were determined by unpaired Student’s *t* test. Statistical significance is displayed as *P < 0.05; **P < 0.01; ***P < 0.001.

## Supplementary information

Supplementary figures 1-5

Supplementary table 1

Unprocessed original images of gels and western blots

Supplementary figure legends

## Data Availability

The RNA-seq data reported in this paper have been deposited in the NCBI Sequence Read Archive (SRA) under accession number PRJNA1132806.
