# Supplementary figures and images for: Critical and differential roles of eIF4A1 and eIF4A2 in B-cell development and function
Source: Cell Mol Immunol. 2024 Nov 8;22(1):40–53. doi: 10.1038/s41423-024-01234-x (PMC11685474; doi:10.1038/s41423-024-01234-x)

**Figure S1**

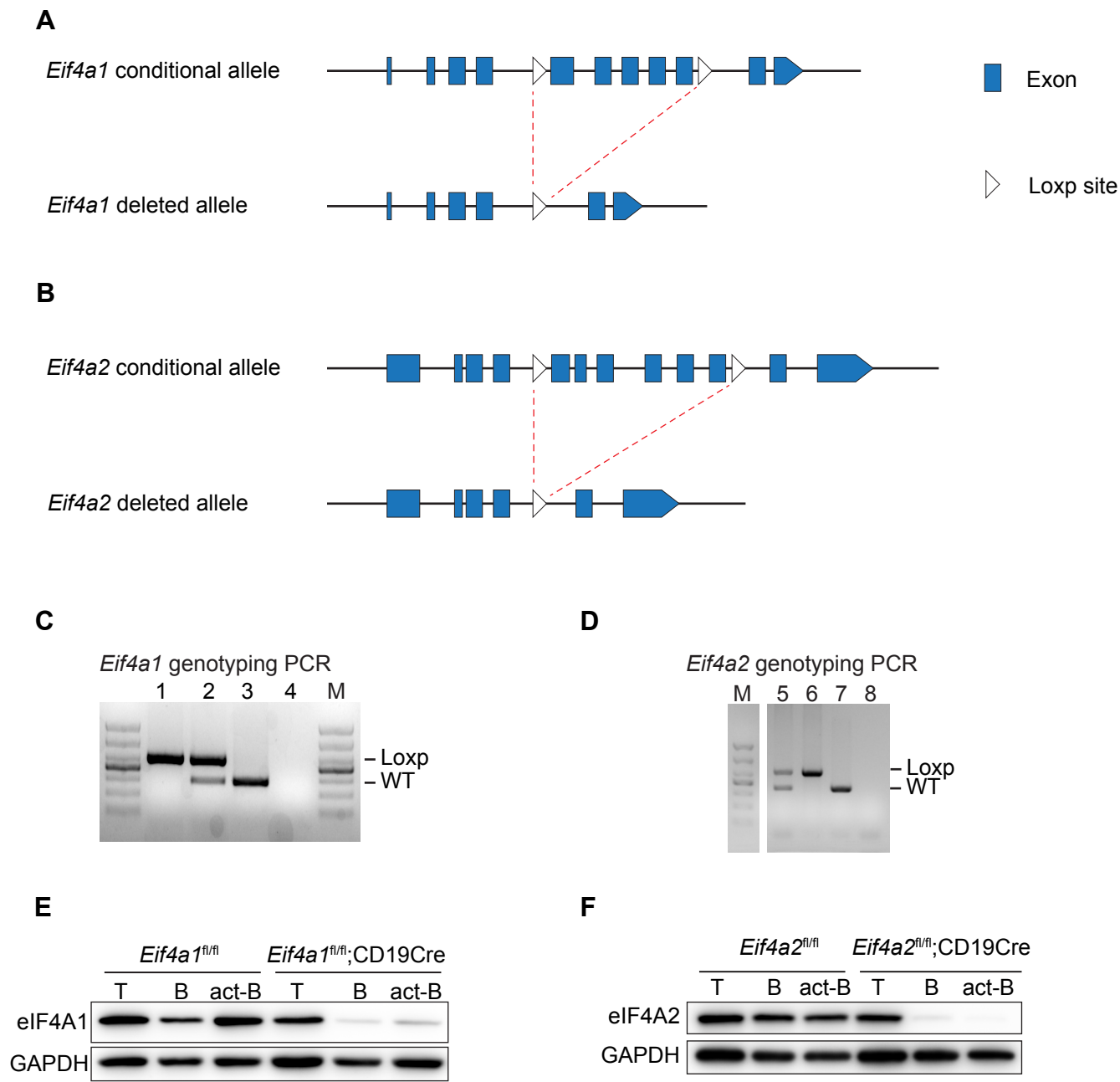

**Figure S2**

**A**

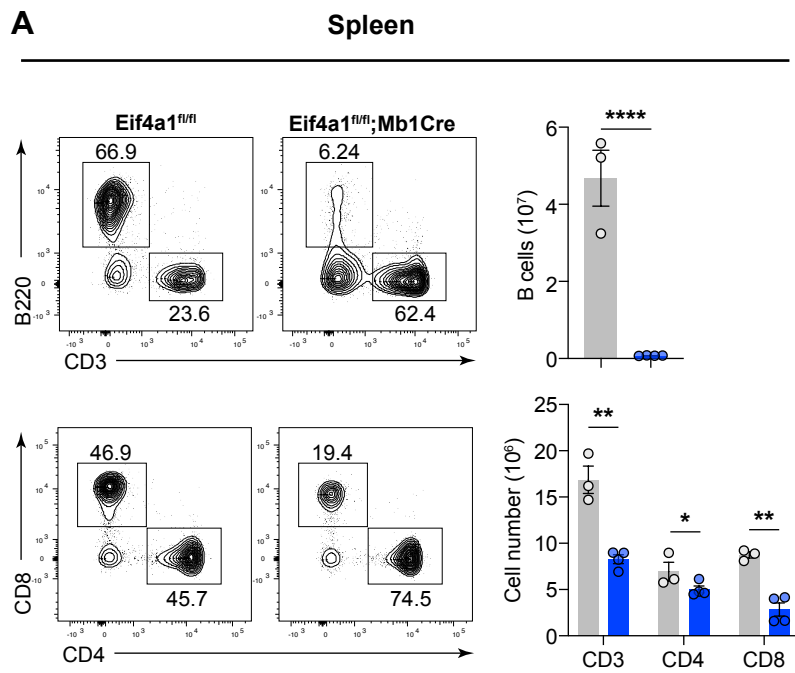

**B**

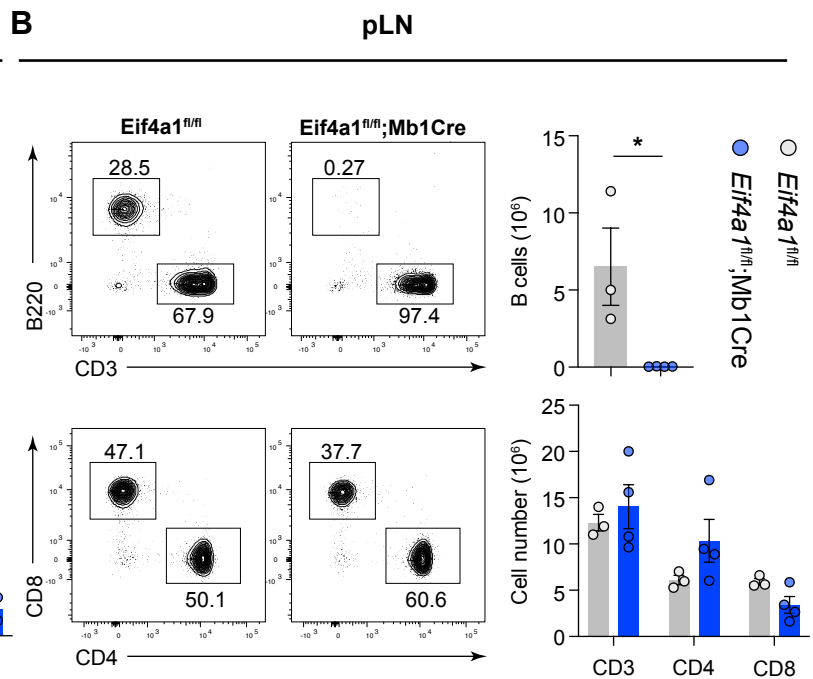

**C**

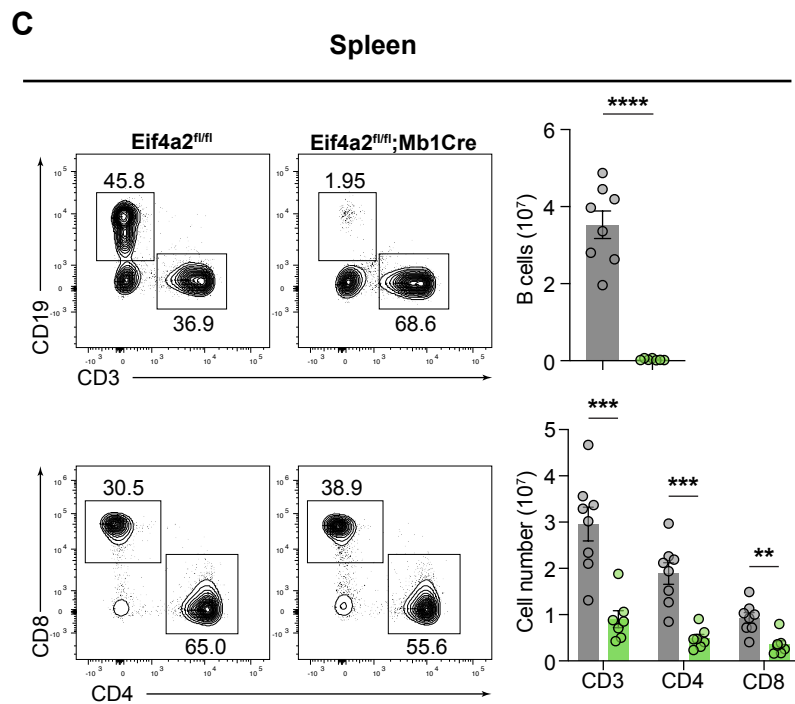

**D**

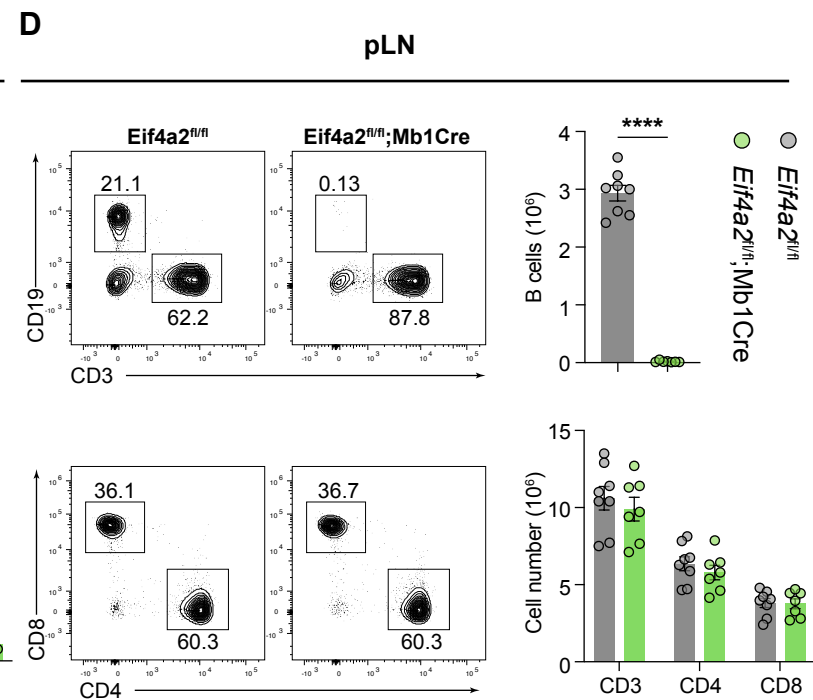

Figure S3

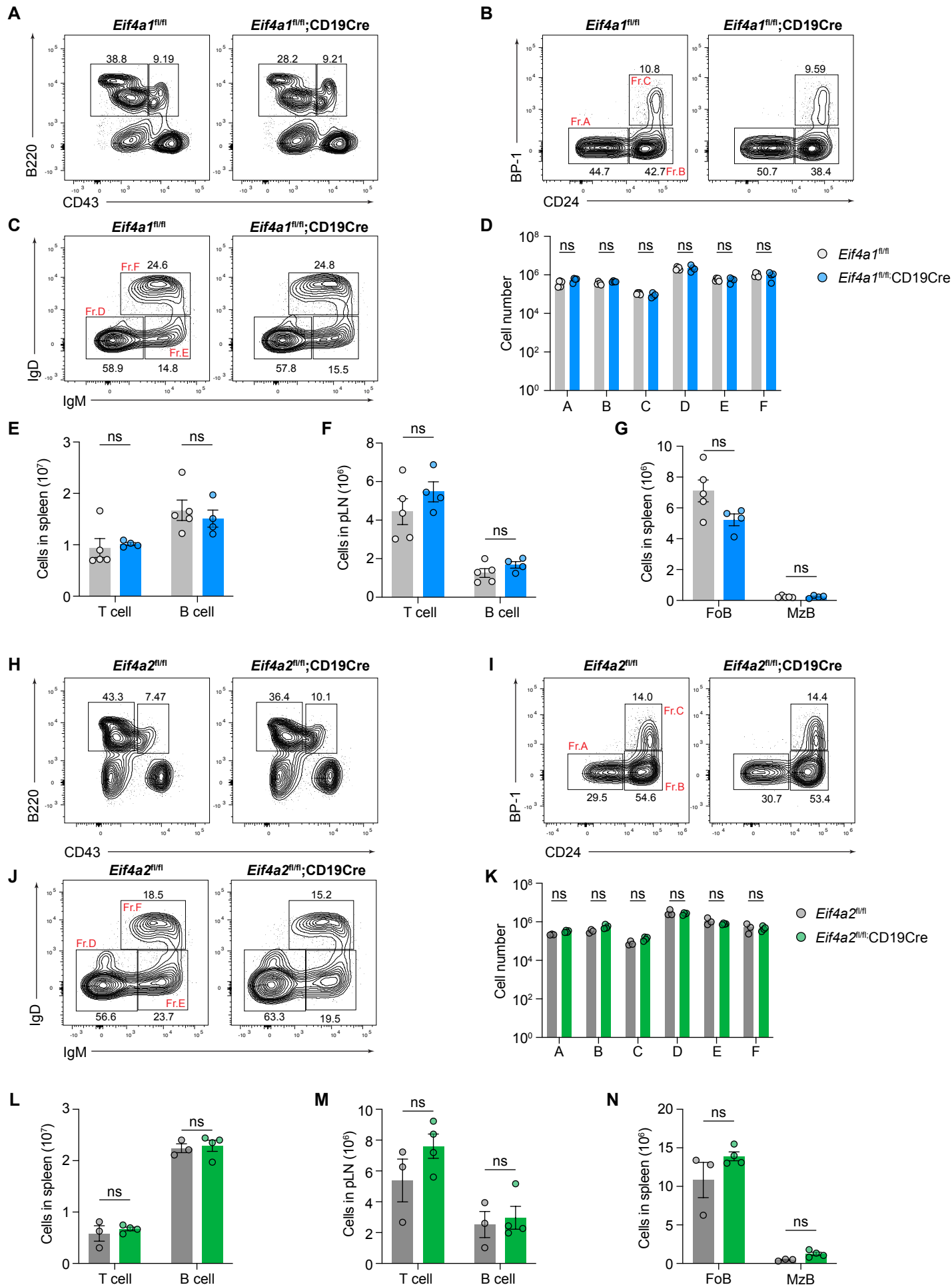

Figure S4

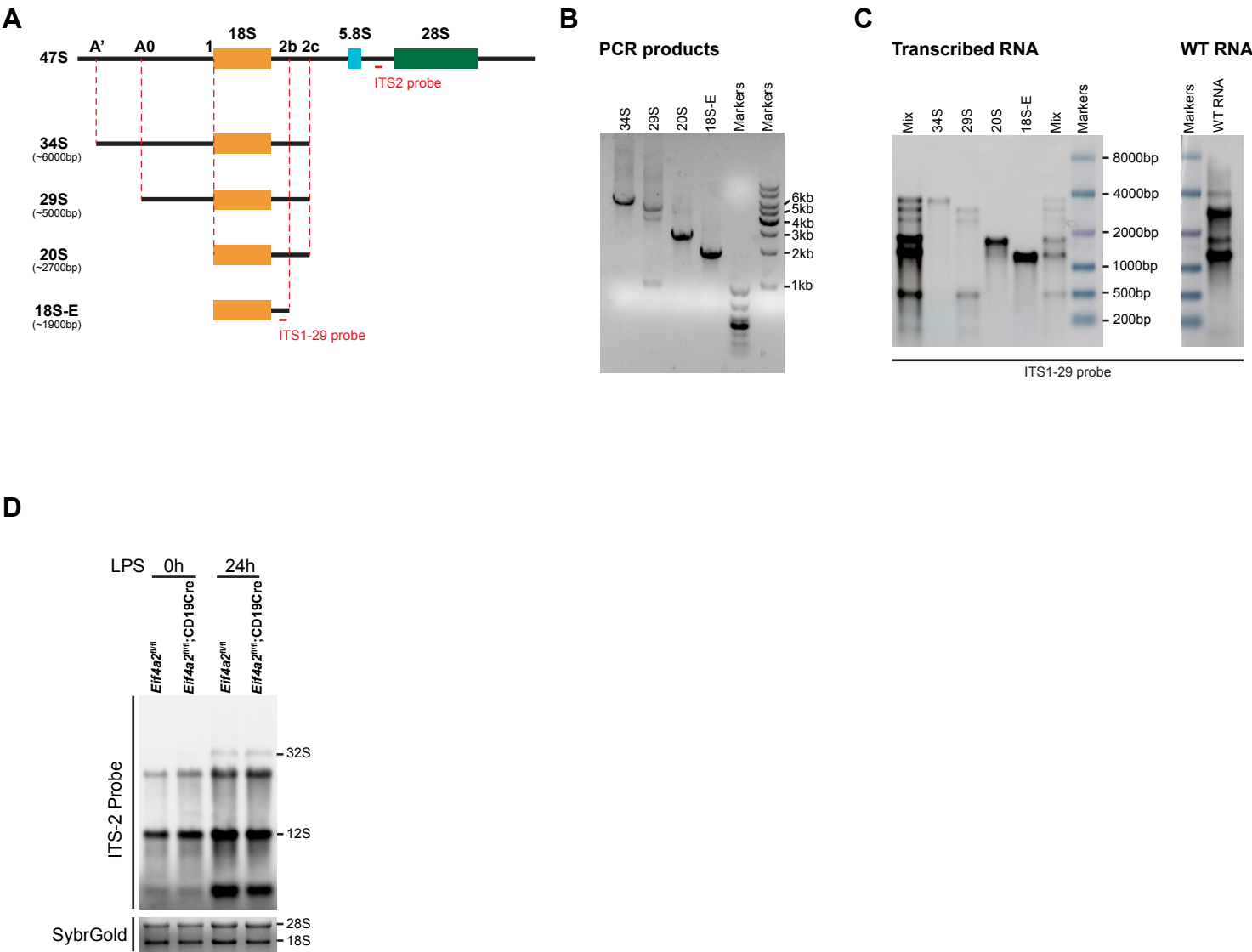

Figure S5

eIF4A1

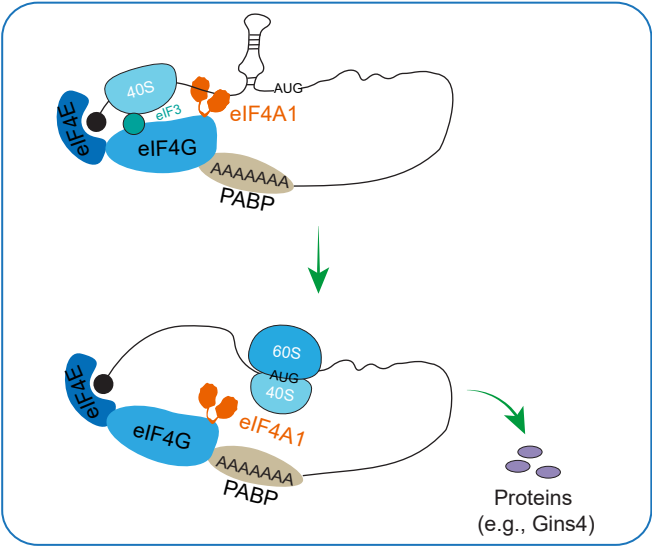

eIF4A2

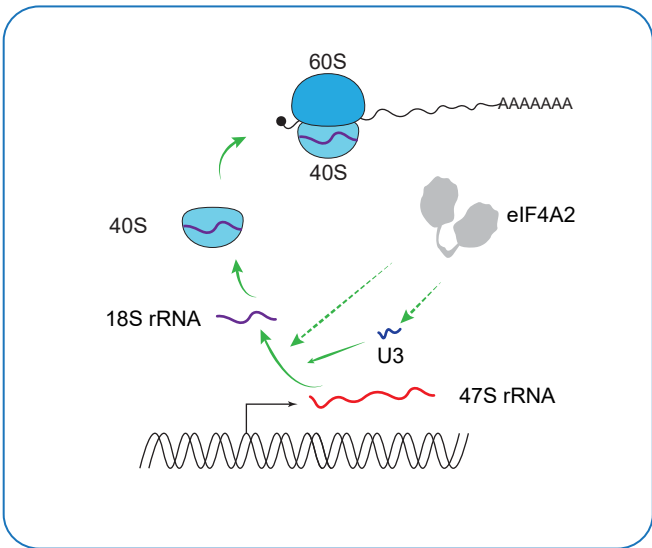

Supplement: Supplementary file 1 — Supplementary figures 1-5 [file 41423_2024_1234_MOESM1_ESM.pdf]
