# Supplementary material for: Critical and differential roles of eIF4A1 and eIF4A2 in B-cell development and function
Source: Cell Mol Immunol. 2024 Nov 8;22(1):40–53. doi: 10.1038/s41423-024-01234-x (PMC11685474; doi:10.1038/s41423-024-01234-x)

Figure 1C

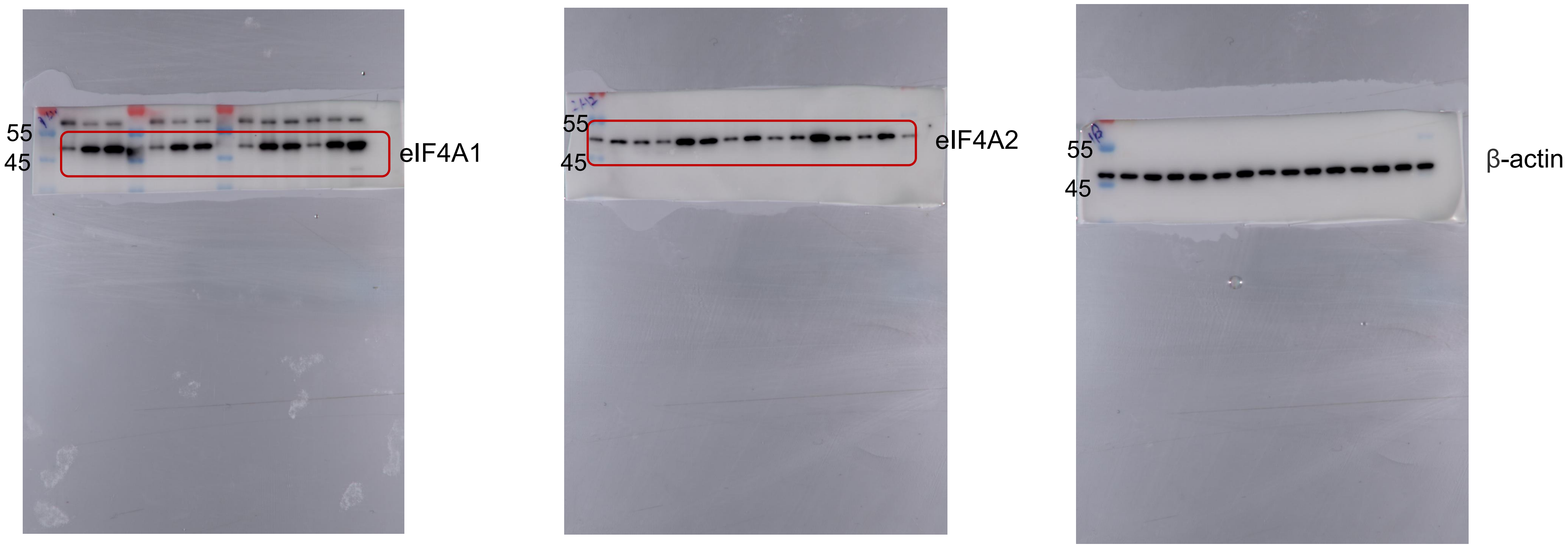

Figure 4A

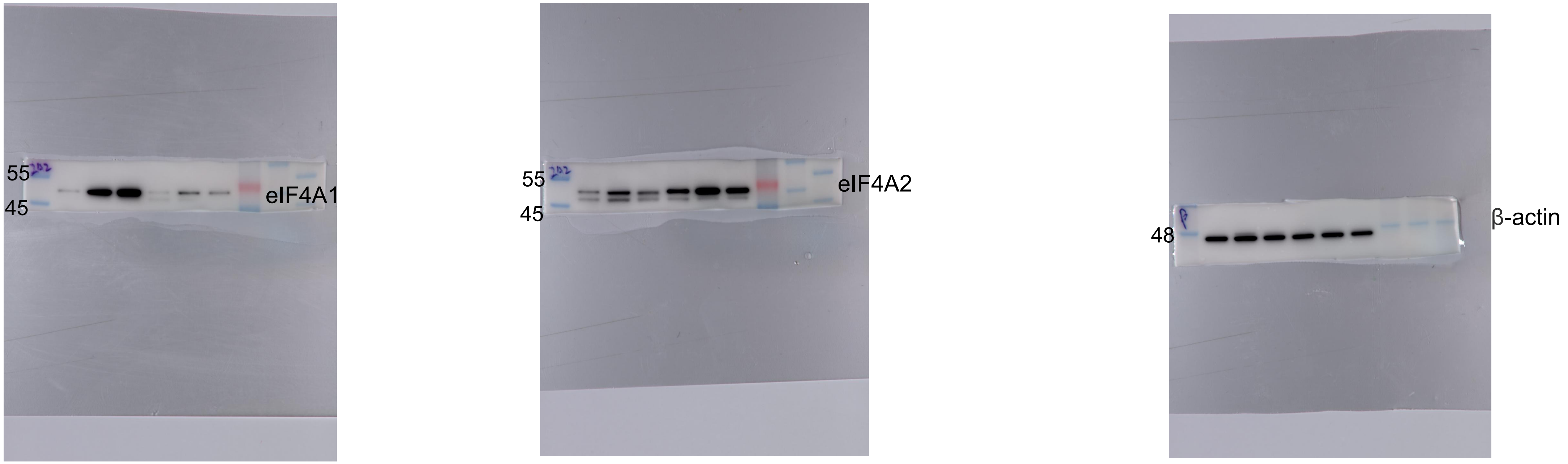

Figure 4B

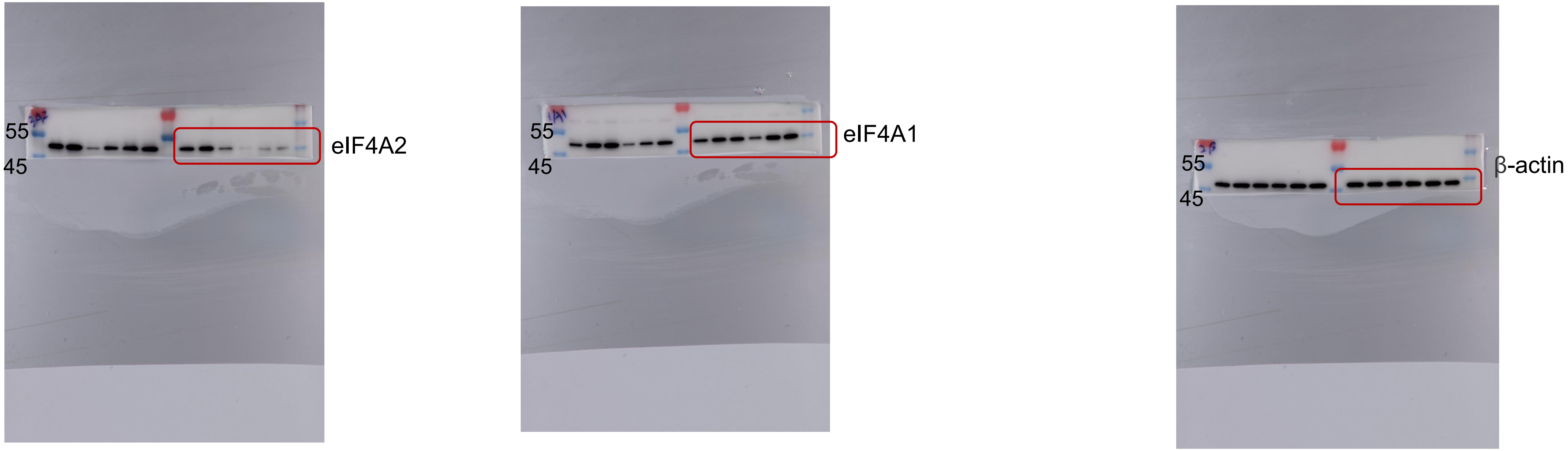

Figure 5D

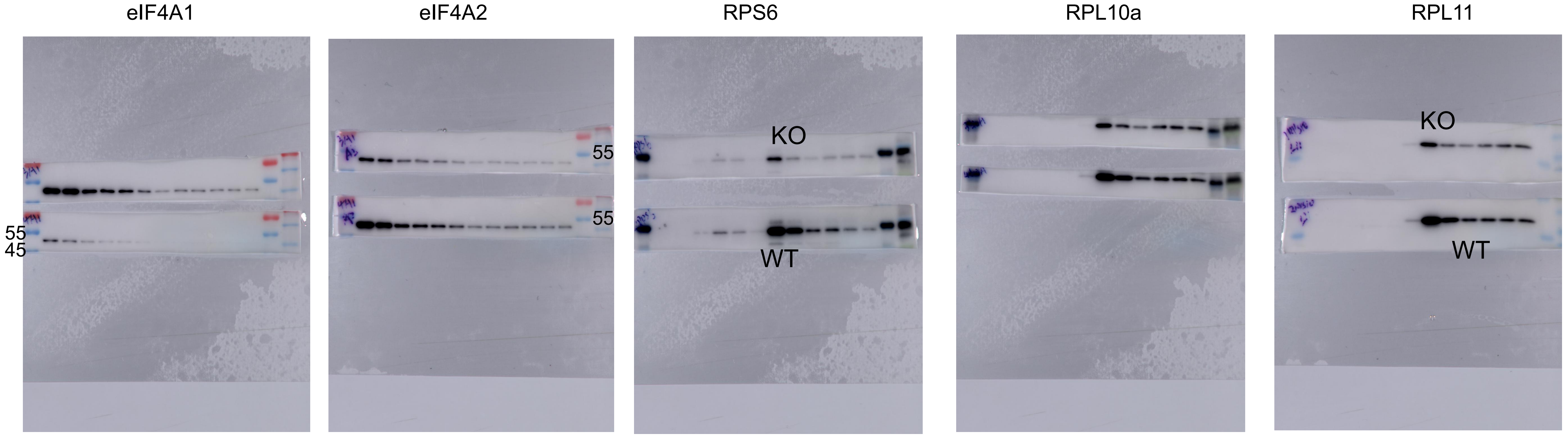

Figure 5E

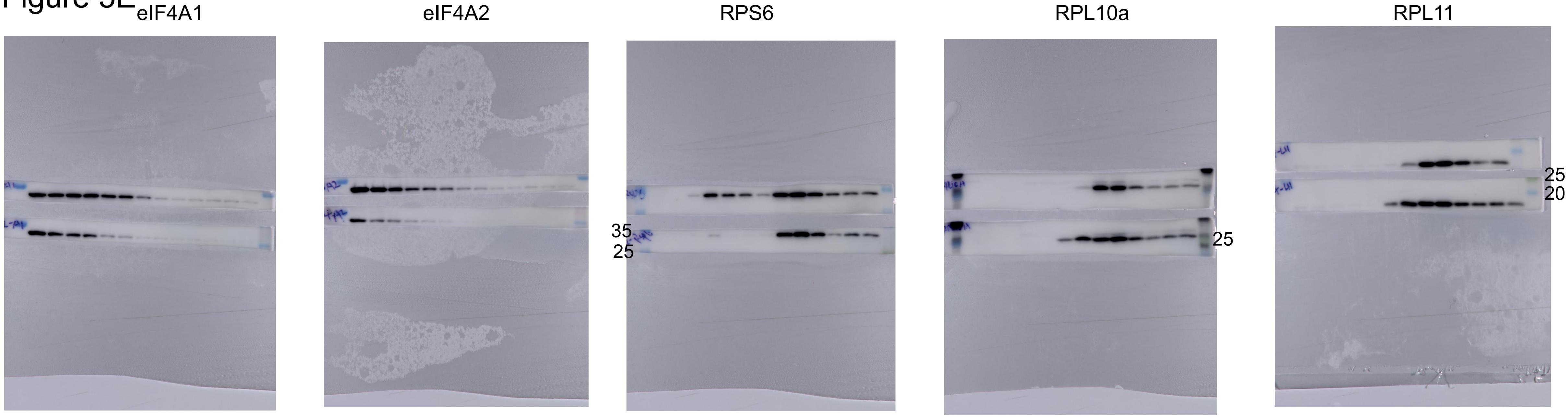

Figure 6A

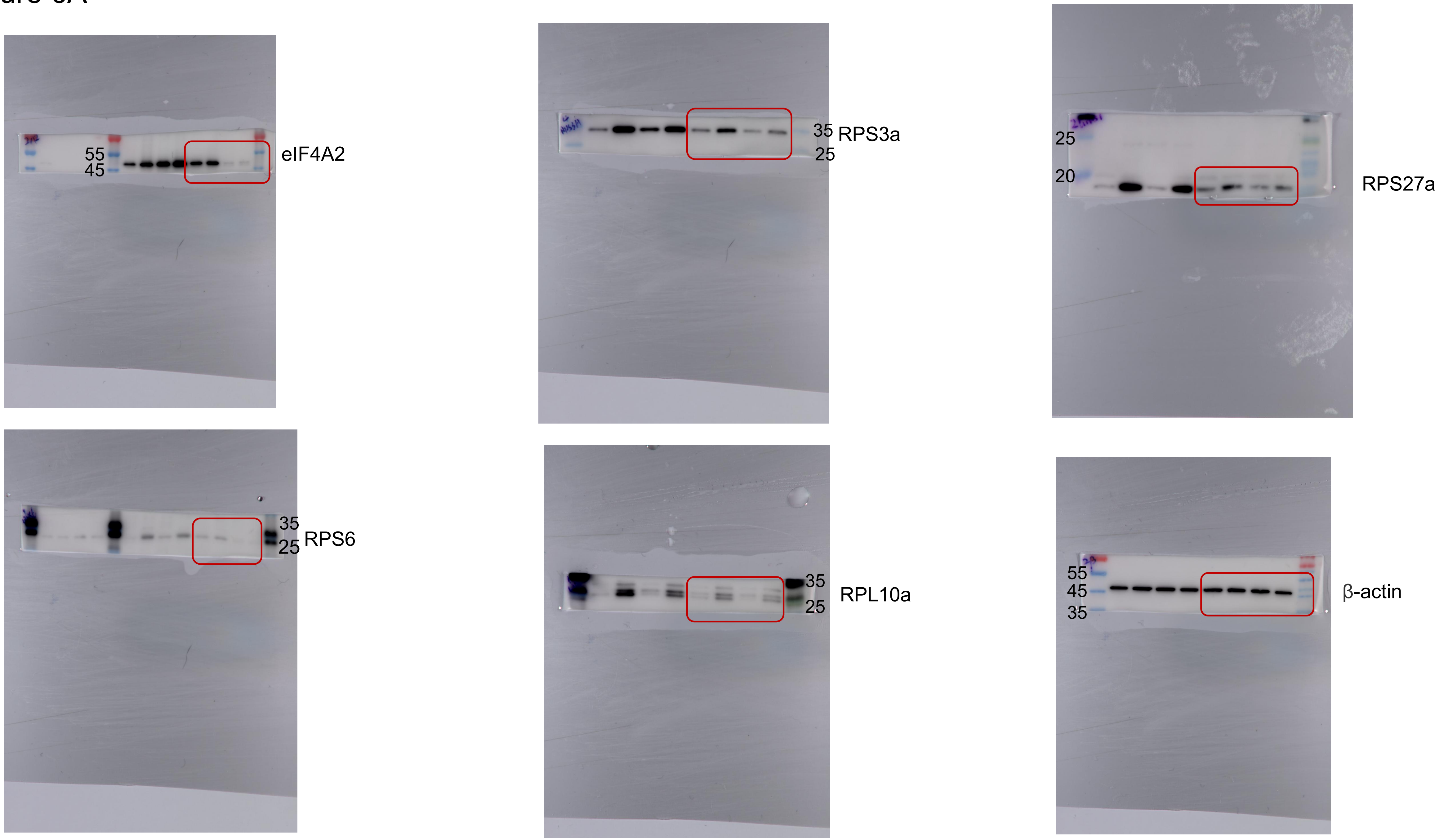

55
45
eIF4A2
35
RPS3a
25
25
20
RPS27a
35
25
RPS6
35
25
RPL10a
55
45
35
β-actin

Figure 6E

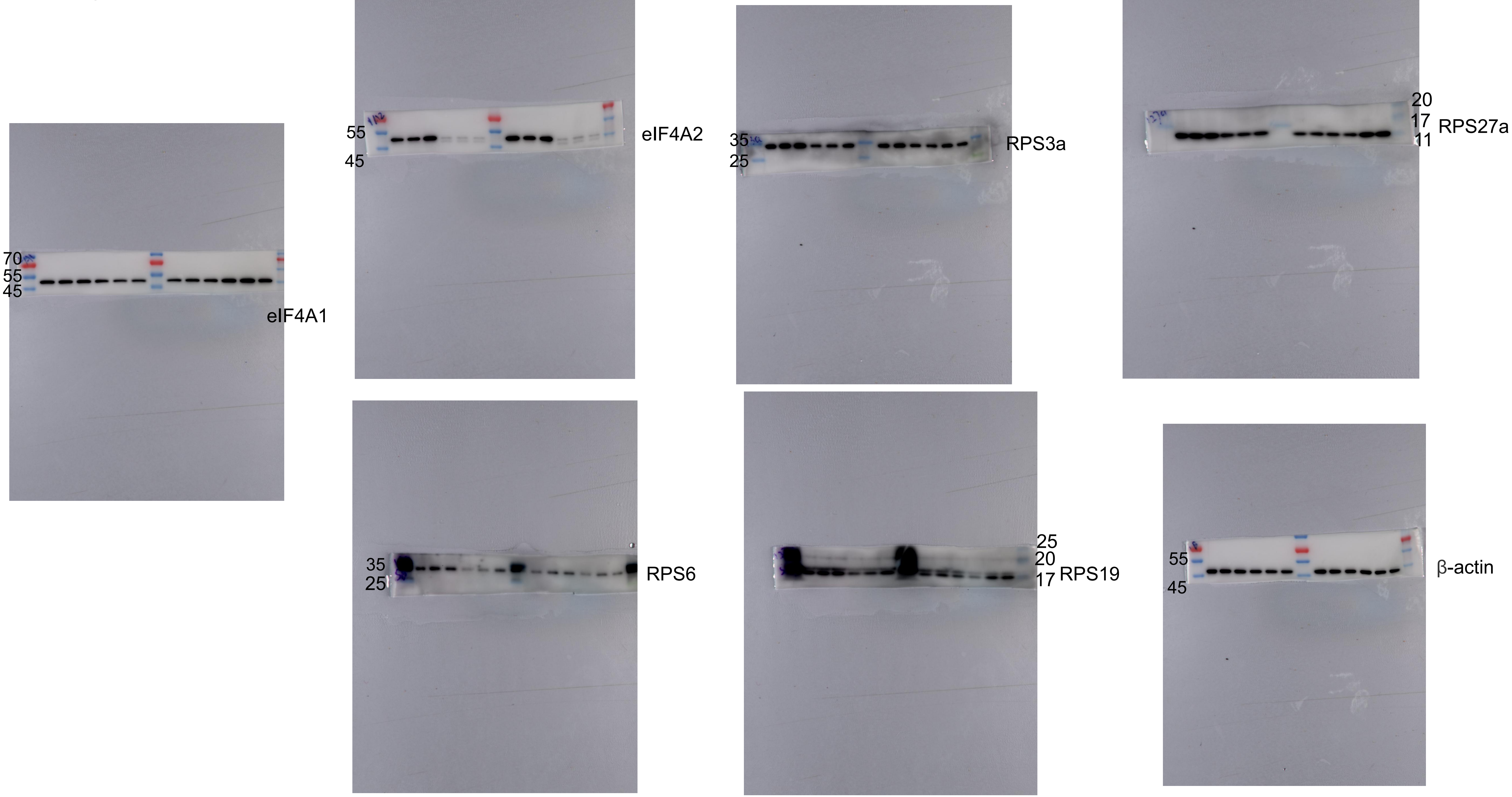

70
55
45
eIF4A1
55
45
eIF4A2
35
25
RPS3a
20
17
11
RPS27a
35
25
RPS6
25
20
17
RPS19
55
45
β-actin

Figure 6G

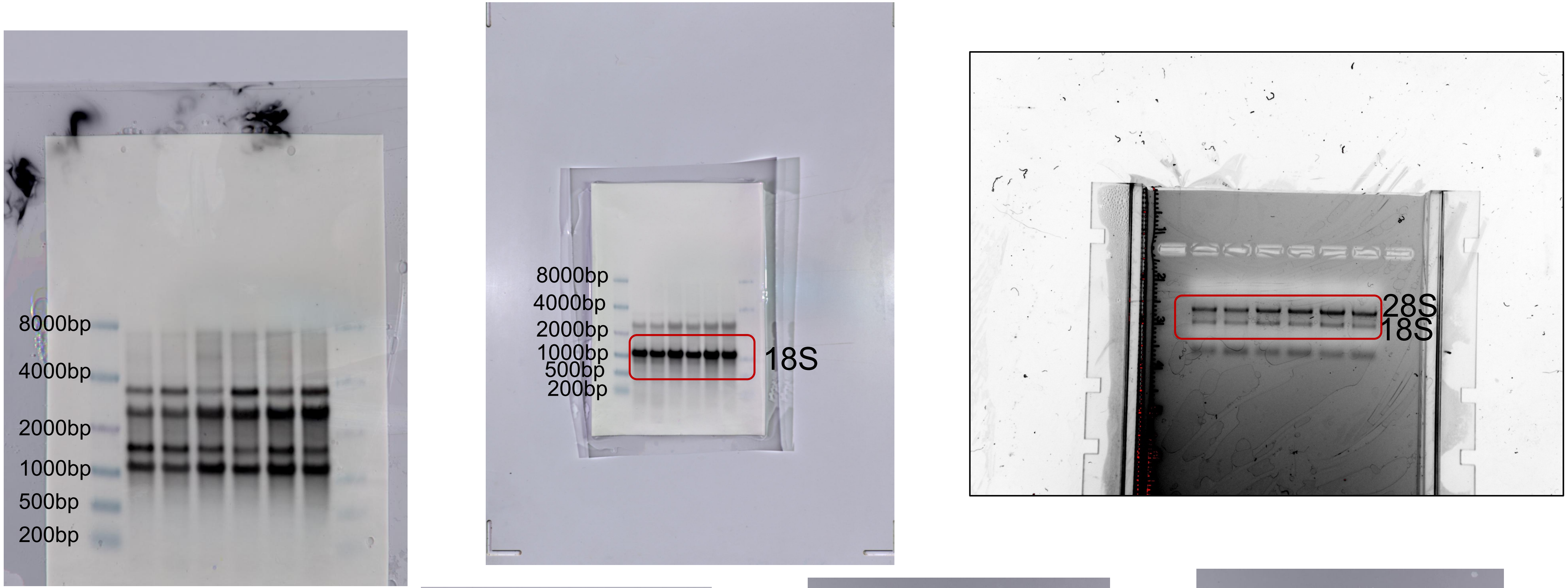

Figure 6H

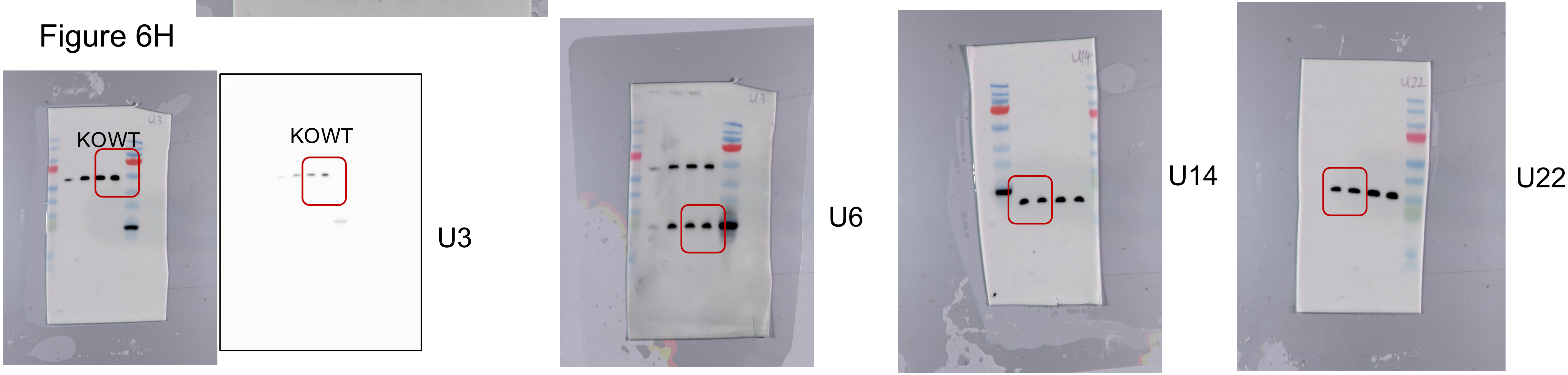

Figure 7E

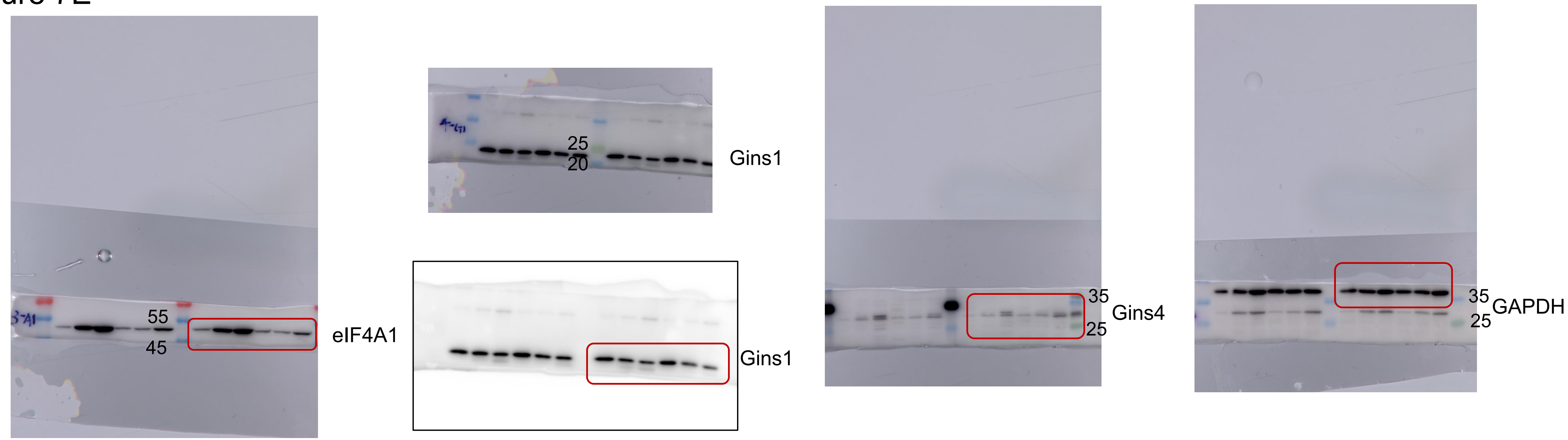

Figure 7I

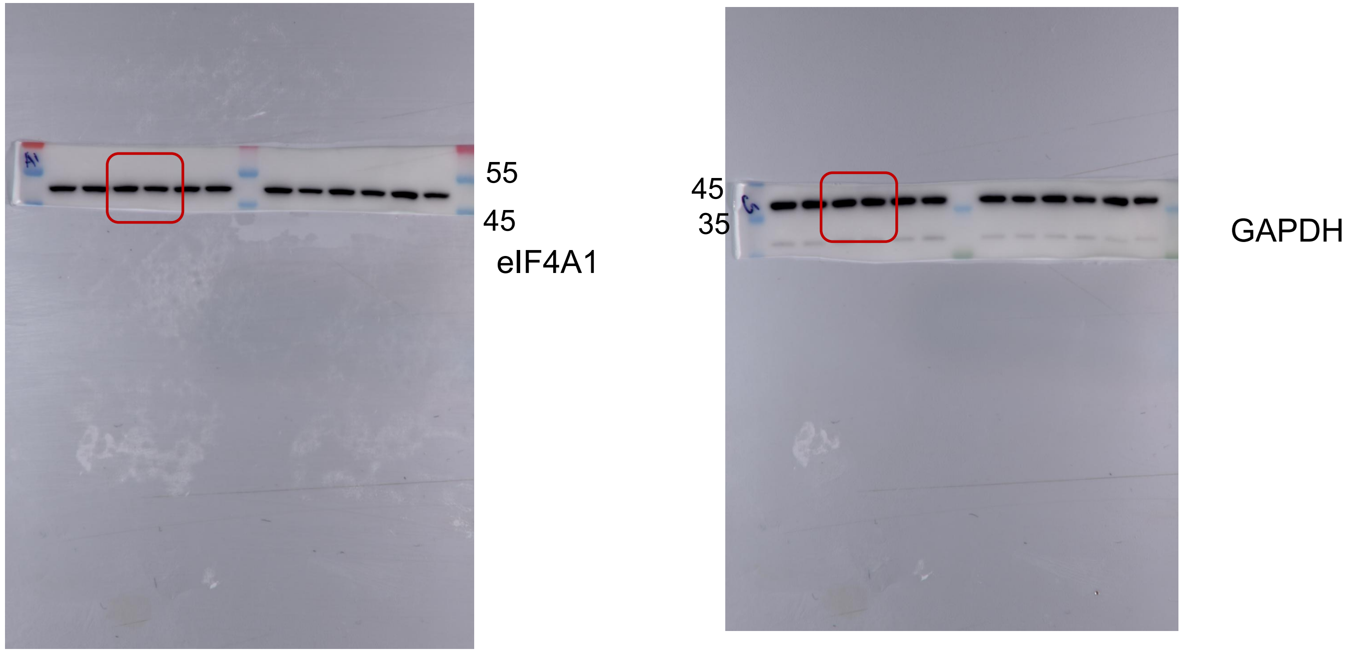

Figure 8A

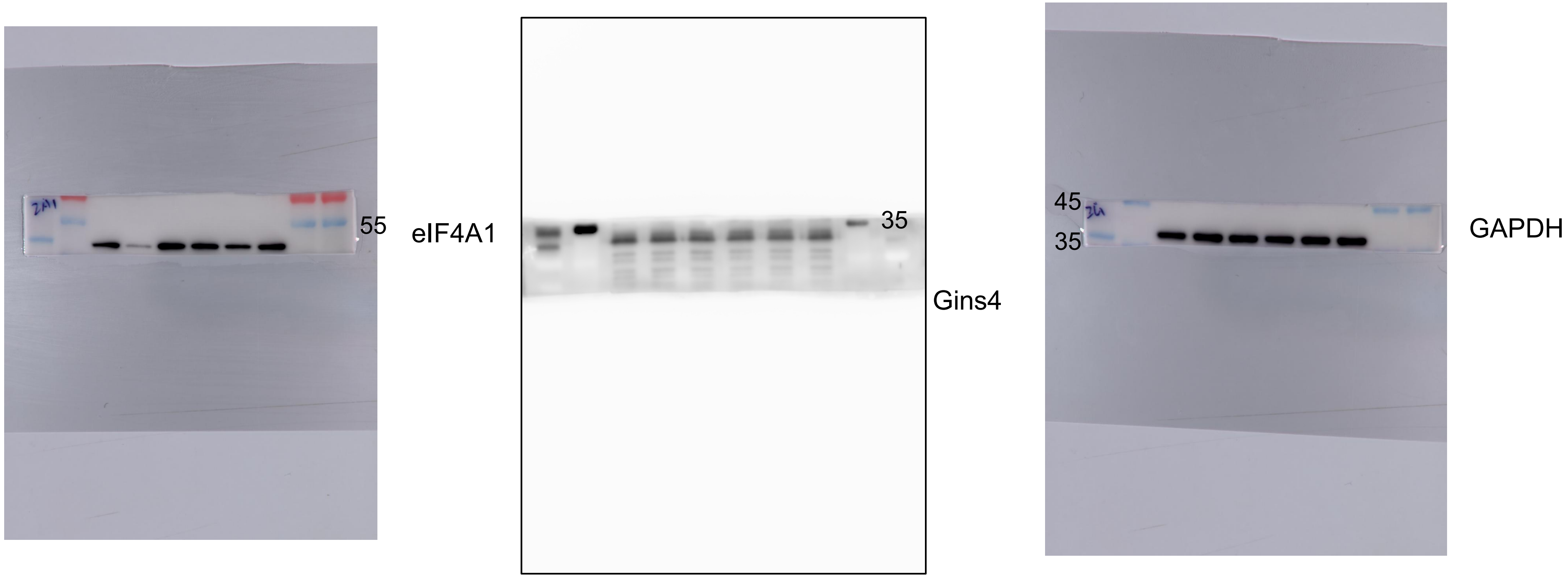

Figure 8D

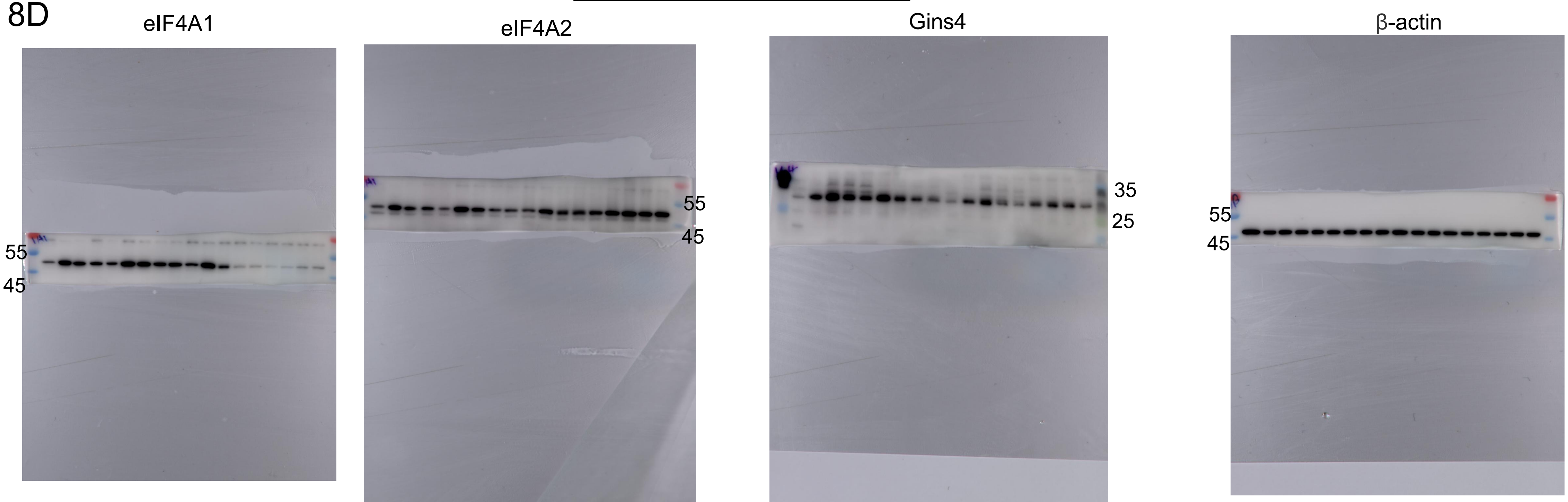

Figure S1C

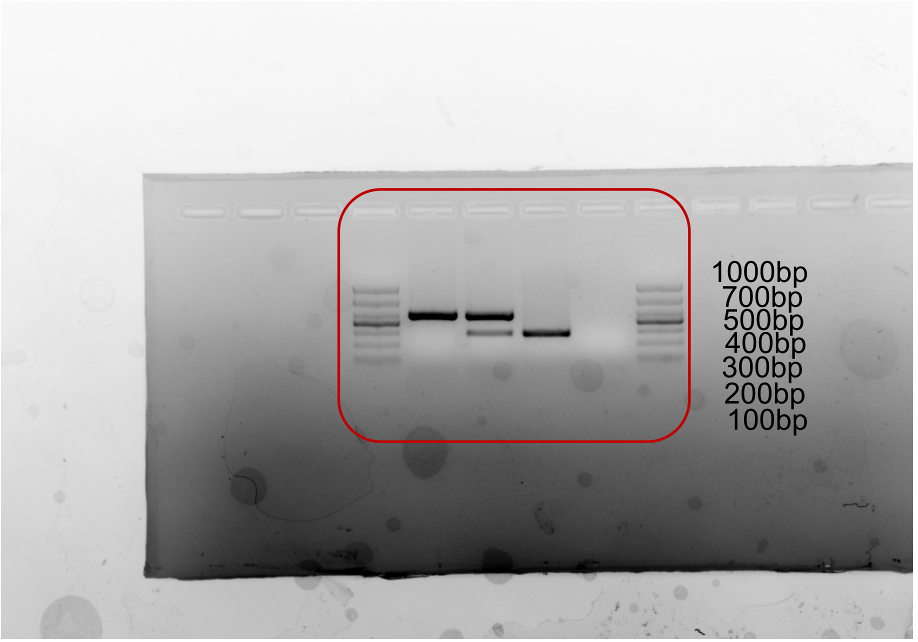

Figure S1D

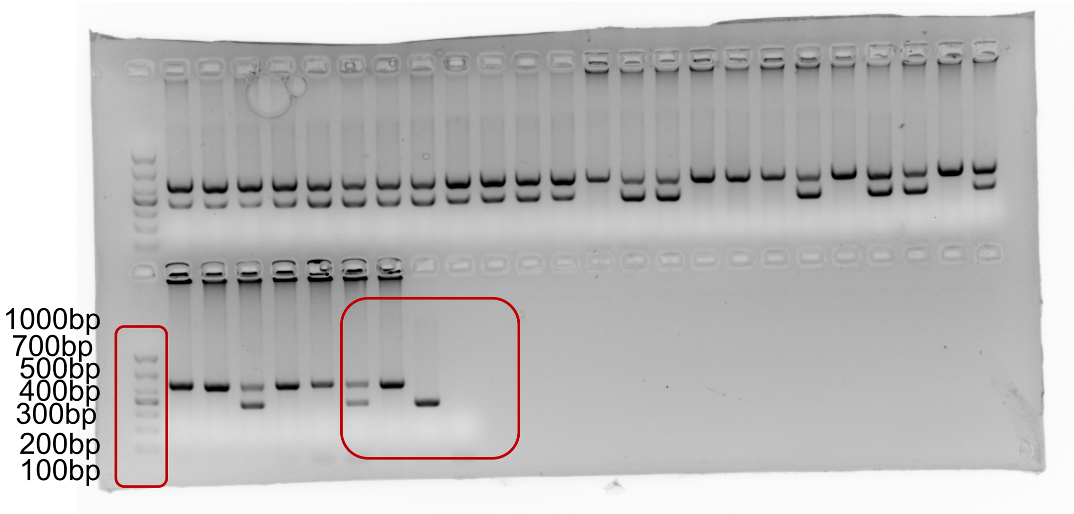

Figure S1E

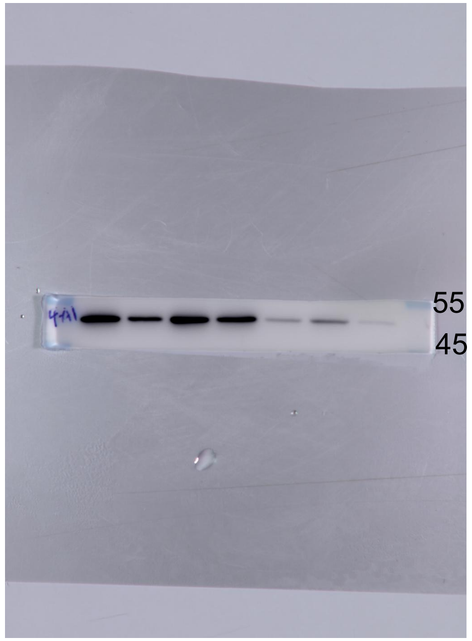

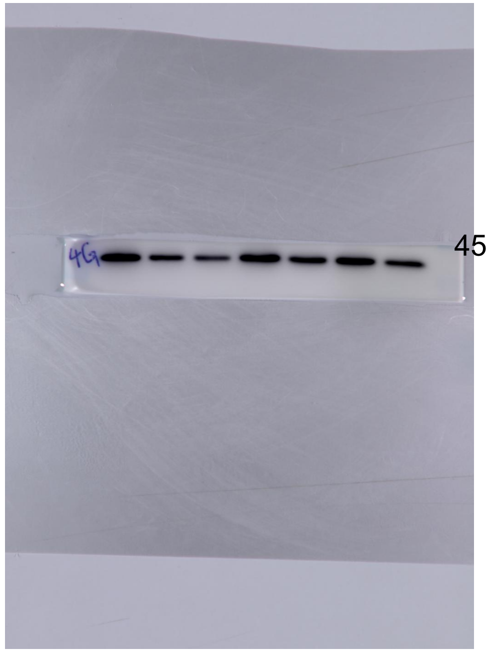

Figure S1F

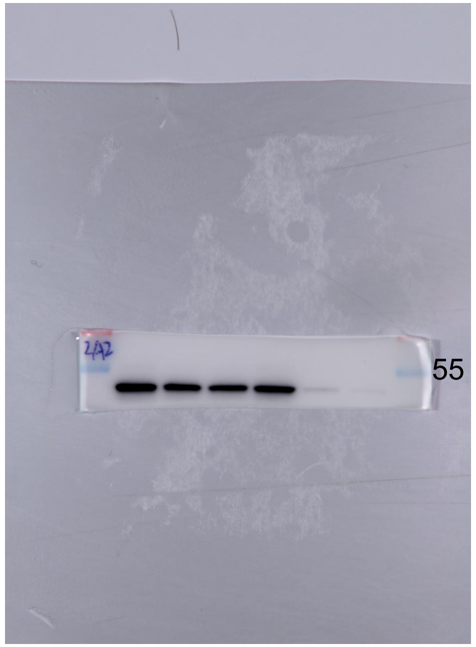

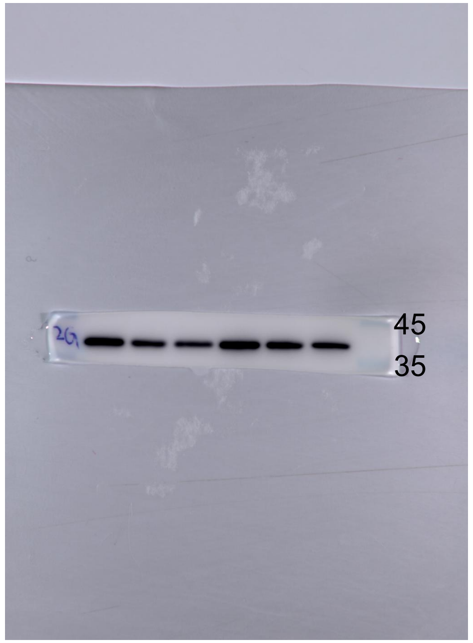

Figure S4B

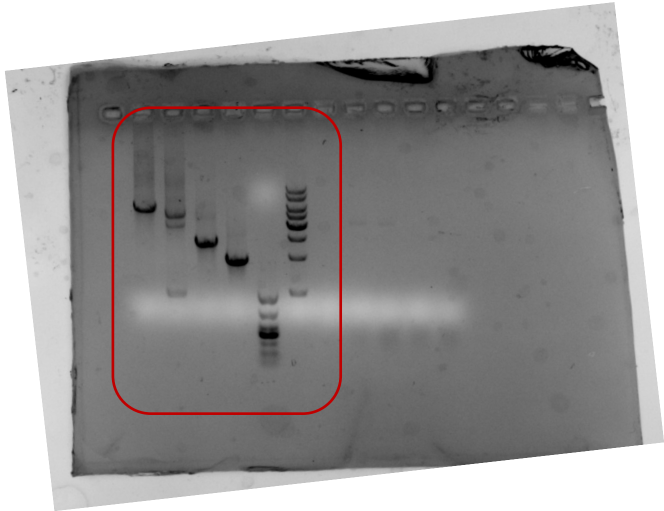

Figure S4C

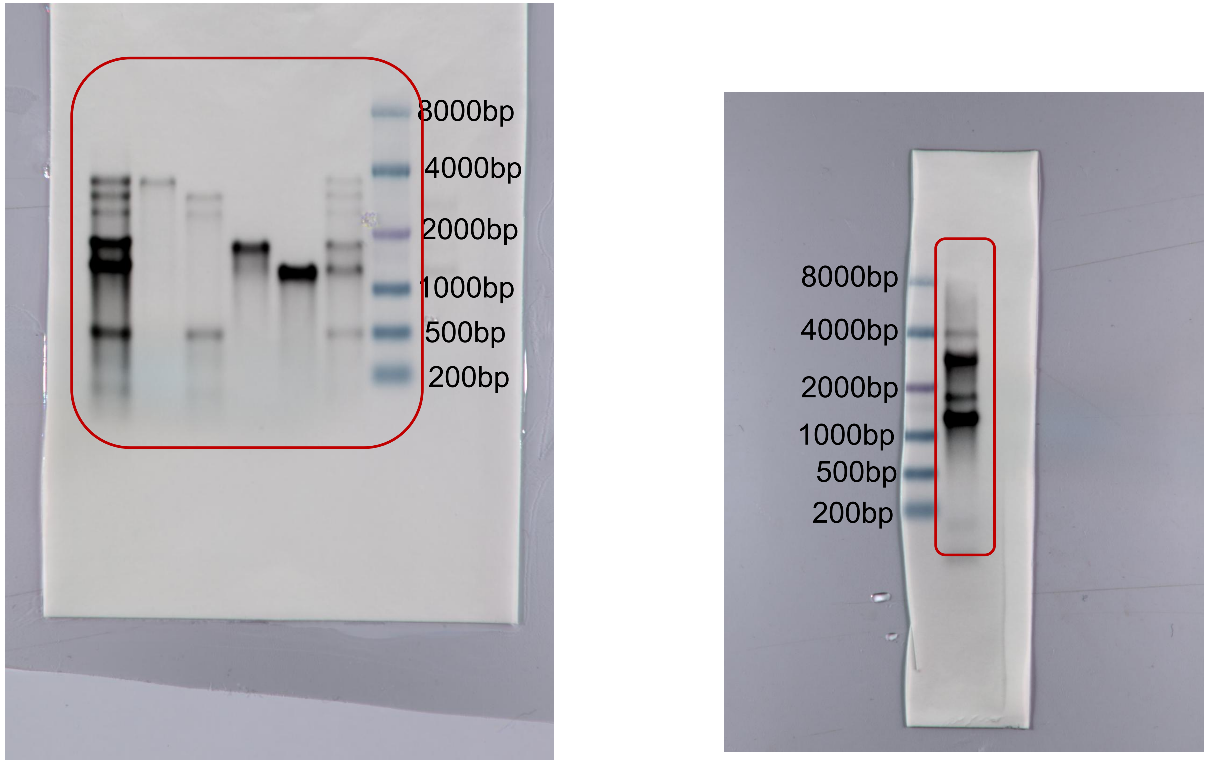

Figure S4D

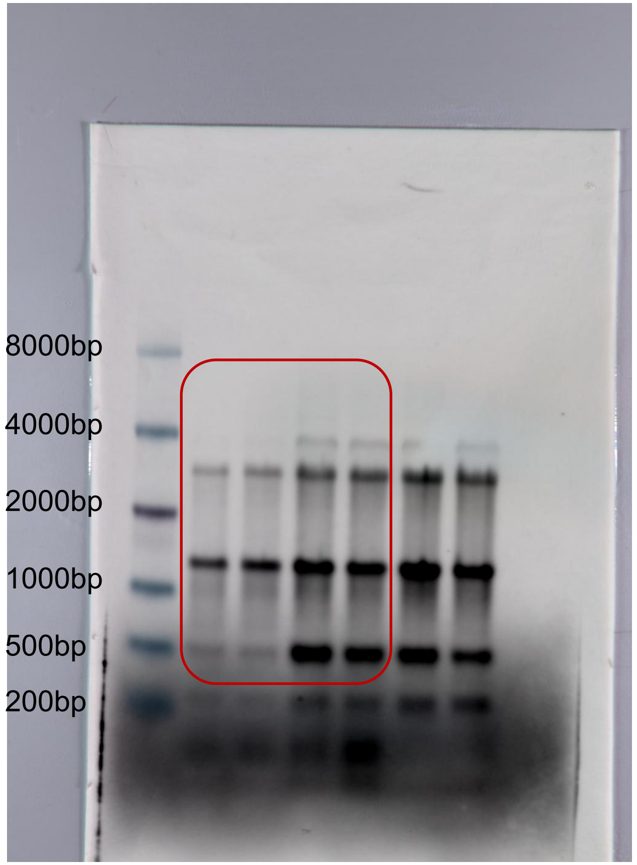

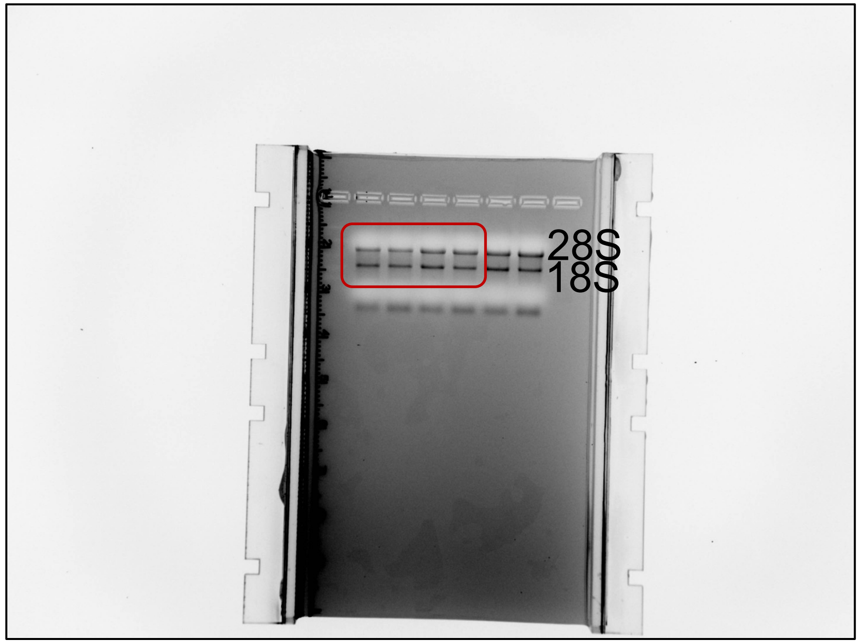

Supplement: Supplementary file 3 — Unprocessed original images of gels and western blots [file 41423_2024_1234_MOESM3_ESM.pdf]
