# Supplementary material for: Critical and differential roles of eIF4A1 and eIF4A2 in B-cell development and function
Source: Cell Mol Immunol. 2024 Nov 8;22(1):40–53. doi: 10.1038/s41423-024-01234-x (PMC11685474; doi:10.1038/s41423-024-01234-x)
Supplement: Supplementary file 4 — Supplementary figure legends [file 41423_2024_1234_MOESM4_ESM.docx]

**Supplementary figure legends**

**Figure S1. Generation of eIF4A1 and eIF4A2 conditional knockout mice**

(**A** and **B**) Scheme of *Eif4a1* (**A**) and *Eif4a2* (**B**) conditional and deleted alleles. (**C** and **D**) Genotyping results for *Eif4a1*^fl/fl^ (**C**) and *Eif4a2*^fl/fl^ (**D**) mice. Lanes 1--4 represent *Eif4a1*^fl/fl^, *Eif4a1*^fl/+^, *Eif4a1*^+/+^ and negative controls, respectively. Lanes 5--8 represent *Eif4a2*^fl/+^, *Eif4a2*^fl/fl^, *Eif4a2*^+/+^ and negative controls. (**E**, **F**) Immunoblot analysis of eIF4A1 (**E**) and eIF4A2 (**F**) protein expression in T, B, and LPS-stimulated B (act-B) cells. (**C**-**F**) The data shown are representative of more than three independent experiments.

**Figure S2. Peripheral B and T cells in *Eif4a1*^fl/fl^;*Mb1Cre* and *Eif4a2*^fl/fl^;*Mb1Cre* mice**

Flow cytometry analysis of T and B cells in the spleen (**A, C**) and peripheral lymph nodes (pLNs, **B, D**) of *Eif4a1*^fl/fl^, *Eif4a1*^fl/fl^;*Mb1Cre* (**A, B**), *Eif4a2*^fl/fl^, and *Eif4a2*^fl/fl^;*Mb1Cre* (**C, D**) mice. Each symbol represents an individual mouse. The error bars represent the standard errors of the means (SEMs). ns, not significant; *p < 0.05, **p<0.01 and ***p<0.001. The data shown are representative of three independent experiments.

**Figure S3. B-cell development in *Eif4a1*^fl/fl^;CD19Cre and *Eif4a2*^fl/fl^;CD19Cre mice**

(**A**-**D, H-K**) Flow cytometry analysis of B lineage cells in the bone marrow of *Eif4a1*^fl/fl^, *Eif4a1*^fl/fl^;CD19Cre (**A**-**D**), *Eif4a2*^fl/fl^, and *Eif4a2*^fl/fl^;CD19Cre (**H**-**J**) mice. (**A-C, H-J**) Representative FACS plots. (**D, K**) Summary of the number of cells in each fraction. (**E, F, L, M**) Summary of T and B cells in the spleen (**E, L**) and peripheral lymph nodes (**F, M**). (**G, N**) Summary of follicular B (FoB) and marginal zone B (MzB) cells in the spleen. (**G**). Each symbol represents an individual mouse. The error bars represent the standard errors of the means (SEMs). ns, not significant; *p < 0.05, **p<0.01 and ***p<0.001. The data shown are representative of three independent experiments.

**Figure S4. Northern blot analysis of 18S and 28S rRNA maturation.**

(**A**) Scheme of ribosomal DNA (rDNA) fragments and northern blot probes. (**B**) Agarose gel analysis of rDNA fragments. (**C**) Northern blot analysis of RNA transcribed from rDNA (transcribed RNA) and total RNA from WT B cells stimulated with LPS for 24 hours (WT RNA) via the ITS1-29 probe. (**D**) Northern blot analysis of total RNA from WT B cells stimulated with LPS for the indicated durations via the ITS-2 probe. Notably, Eif4A2 deficiency has no effect on 28S maturation**.** The data shown are representative of three independent experiments.

**Figure S5. Graphic summary of eIF4A1 and eIF4A2 functions in translation**

The primary role of eIF4A1 is to unwind secondary structures in the 5' UTR of mRNA, which is essential for the preinitiation complex (PIC, represented by the 40S ribosome subunit) to scan the 5’UTR to find the start codon. This is followed by recruitment of the 60S ribosome subunit and assembly of the 80S ribosome, which is now poised for translation initiation. Among the genes related to eIF4A1 are Gins4, which encodes a critical subunit of the Cdc45-MCM-GINS (CMG) complex that is responsible for unwinding DNA during the S phase of the cell cycle (left panel). eIF4A2 plays critical roles in multiple steps of 18S rRNA processing and maturation, including regulating the expression of U3 snoRNAs, thereby controlling the biogenesis of the 40S ribosome subunit (right panel).
